# MHC Ib molecule Qa-1 presents *Mycobacterium tuberculosis* peptide antigens to CD8^+^ T cells and contributes to protection against infection

**DOI:** 10.1371/journal.ppat.1006384

**Published:** 2017-05-05

**Authors:** Yao Bian, Shaobin Shang, Sarah Siddiqui, Jie Zhao, Simone A. Joosten, Tom H. M. Ottenhoff, Harvey Cantor, Chyung-Ru Wang

**Affiliations:** 1 Department of Microbiology and Immunology, Feinberg School of Medicine Northwestern University, Chicago, Illinois, United States of America; 2 Department of Infectious Diseases, Leiden University Medical Center, Leiden, The Netherlands; 3 Department of Cancer Immunology and Virology, Dana-Farber Cancer Institute, Department of Microbiology and Immunobiology, Division of Immunology, Harvard Medical School Boston, Massachusetts, United States of America; Portland VA Medical Center, Oregon Health and Science University, UNITED STATES

## Abstract

A number of nonclassical MHC Ib molecules recognizing distinct microbial antigens have been implicated in the immune response to *Mycobacterium tuberculosis* (Mtb). HLA-E has been identified to present numerous Mtb peptides to CD8^+^ T cells, with multiple HLA-E-restricted cytotoxic T lymphocyte (CTL) and regulatory T cell lines isolated from patients with active and latent tuberculosis (TB). In other disease models, HLA-E and its mouse homolog Qa-1 can act as antigen presenting molecules as well as regulators of the immune response. However, it is unclear what precise role(s) HLA-E/Qa-1 play in the immune response to Mtb. In this study, we found that murine Qa-1 can bind and present Mtb peptide antigens to CD8^+^ T effector cells during aerosol Mtb infection. Further, mice lacking Qa-1 (Qa-1^-/-^) were more susceptible to high-dose Mtb infection compared to wild-type controls, with higher bacterial burdens and increased mortality. The increased susceptibility of Qa-1^-/-^ mice was associated with dysregulated T cells that were more activated and produced higher levels of pro-inflammatory cytokines. T cells from Qa-1^-/-^ mice also had increased expression of inhibitory and apoptosis-associated cell surface markers such as CD94/NKG2A, KLRG1, PD-1, Fas-L, and CTLA-4. As such, they were more prone to cell death and had decreased capacity in promoting the killing of Mtb in infected macrophages. Lastly, comparing the immune responses of Qa-1 mutant knock-in mice deficient in either Qa-1-restricted CD8^+^ T_regs_ (Qa-1 D227K) or the inhibitory Qa-1-CD94/NKG2A interaction (Qa-1 R72A) with Qa-1^-/-^ and wild-type controls indicated that both of these Qa-1-mediated mechanisms were involved in suppression of the immune response in Mtb infection. Our findings reveal that Qa-1 participates in the immune response to Mtb infection by presenting peptide antigens as well as regulating immune responses, resulting in more effective anti-Mtb immunity.

## Author summary

The disease tuberculosis (TB) is caused by the microbe *Mycobacterium tuberculosis* (Mtb), and remains a major public health concern. More research is needed to understand the diverse immune responses against Mtb to develop better vaccines. Mouse Qa-1 and its human counterpart HLA-E are nonclassical MHC I molecules that can activate or inhibit immune responses in a variety of diseases. However, their role during the immune response to Mtb remains unknown. We found that Qa-1 can present Mtb peptides to activate CD8^+^ T effector cells during aerosol Mtb infection. Further, Mtb-infected mice that lacked Qa-1 (Qa-1^-/-^) had higher numbers of bacteria and died more often than infected mice that expressed Qa-1 (Qa-1^+/+^). The lack of Qa-1 results in over-activation of the immune response upon infection, which is less efficient in controlling Mtb. Using mice expressing different mutant forms of Qa-1, we showed that Qa-1 can regulate immune responses against Mtb through the interaction with inhibitory CD94/NKG2A receptors as well as the activation of regulatory CD8^+^ T cells. We believe our study sheds light on the diverse mechanisms at play in generating protective immune responses against Mtb and will inform future mouse and human studies.

## Introduction

As the causative agent of tuberculosis (TB), *Mycobacterium tuberculosis* (Mtb) is a continuing global public health concern that kills approximately 2 million people annually, with an estimated one-third of the world’s population infected with Mtb [1]. The emergence of multidrug-resistant Mtb, co-infection with HIV, and the limited efficacy of the *Mycobacterium bovis* Bacillus Calmette-Guérin (BCG) vaccine compound the need for further research on the immune responses to pulmonary Mtb infection, toward the development of more effective TB vaccines.

Optimal protection against Mtb requires both CD4^+^ and CD8^+^ T cell responses. Most Mtb-specific CD4^+^ T cells produce Th1-type cytokines, including IFN-γ, TNF-α, IL-12, and IL-2 [2]. Similarly, Mtb-specific CD8^+^ T cells are also potent producers of IFN-γ and TNF-α, cytokines crucial to anti-Mtb immunity [3, 4]. While current Mtb subunit vaccine development has primarily focused on CD4^+^ and MHC Ia-restricted CD8^+^ T cell responses [5], increasing evidence shows that non-conventional CD8^+^ T cells restricted by MHC Ib molecules can recognize distinct microbial antigens and contribute to protection against Mtb infection [6–8]. Like MHC Ia molecules, MHC Ib molecules can present antigens to CD8^+^ cytotoxic T lymphocytes (CTL). However, MHC Ib molecules are less polymorphic than MHC Ia molecules, making them attractive targets for vaccine development. In particular, MHC Ib molecules CD1, MR1, Qa-1/HLA-E and Qa-2/HLA-G have been implicated in the host immune response against Mtb in mice and/or humans [6–10]. Group 1 CD1-restricted T cells specific to Mtb lipid have been detected in patients with active or latent TB infection and conferred protection against Mtb in human group 1 CD1 transgenic mice [11–15]. MR1-restricted mucosal-associated invariant T cells (MAIT) recognizing vitamin B metabolites were also shown to participate in anti-mycobacterial immunity [16, 17]. In addition, Qa-2-restricted and other nonclassical MHC Ib-restricted CD8^+^ T Cells have been shown to provide protection against Mtb in mice [10]. Recently, 69 Mtb peptides have been shown to have predicted binding affinity to HLA-E, with most of these peptides inducing CD8^+^ T cell proliferation when presented by HLA-E to PBMCs from mycobacterial- responsive donors [8]. Multiple HLA-E-restricted Mtb peptide-specific CTLs from these donors have also been isolated [8, 18–20]. Further, HLA-E tetramers have identified Mtb peptide-specific CD8^+^ T cells in PBMCs of TB patients at the highest frequency during active infection [18], suggesting that HLA-E constitutes an important element in Mtb immunity.

Like other MHC Ib molecules, HLA-E exhibits limited polymorphism; it has 3 protein variants, only 2 of which are detected in the human population with high frequency [21]. HLA-E has a structural and functional mouse homolog, Qa-1 (H2-T23) [22, 23], and both human and mouse molecules have been shown to play diverse roles in the immune system. Both HLA-E and Qa-1 can present peptide antigens from intracellular pathogens such as Epstein Barr virus, cytomegalovirus, and *Salmonella typhimurium* to CD8^+^ T cells, resulting in activation of CTL activity against infected cells [23–25]. HLA-E and Qa-1 also both predominantly bind endogenous peptides derived from the leader sequence of MHC Ia molecules [26, 27]. For Qa-1, this single nonameric peptide is called Qa-1 determinant modifier (Qdm). The Qa-1/Qdm or HLA-E/peptide complex serves as a ligand for CD94/NKG2 receptors, which are expressed mainly on NK cells and a subset of CD8^+^ T cells [28, 29]. CD94 is primarily associated with the NKG2A isoform, forming an inhibitory receptor, but can also be complexed with NKG2C/E, forming activating receptors [23]. Ligation of Qa-1/Qdm with CD94/NKG2A results in inhibition of NK cell cytolytic activity [30]. As the presentation of Qdm and other leader sequence peptides is dependent on transporter associated with antigen processing (TAP), the absence of HLA-E/Qa-1 bound to the endogenous peptides allows NK cells to detect and lyse abnormal cells [31, 32]. CD8^+^ T cell responses to viral infection can also be dampened through the interaction of Qa-1/Qdm with CD94/NKG2A [33–36]. Lastly, Qa-1 is the restriction element for a subset of suppressor CD8^+^ T cells, called Qa-1-restricted CD8^+^ regulatory T cells (CD8^+^ T_reg_) [37]. Qa-1-restricted CD8^+^ T_reg_ cells have been shown to suppress the development of murine experimental autoimmune encephalomyelitis (EAE) and other autoimmune diseases [38–40]. Further, Qa-1-deficient (Qa-1^-/-^) mice developed exaggerated CD4^+^ T cell responses upon viral infection or immunization with self-peptide compared to wild-type mice, due to a lack of Qa-1-restricted CD8^+^ T_regs_ [24, 41]. Although CD8^+^ T_regs_ have not been well-studied in the context of HLA-E, a number of Mtb-specific HLA-E restricted CTL and/or regulatory CD8^+^ T cell clones have been shown to exhibit suppressive activity [8, 18, 20]. In addition, glatiramer acetate induced CD8^+^ T cells from human multiple sclerosis patients have HLA-E-restricted regulatory activity [42]. In summary, HLA-E and Qa-1 can present both self and foreign peptides and interact with various receptors, resulting in both activation and suppression of immune responses through a variety of mechanisms.

Although Mtb peptide-specific, HLA-E restricted CD8^+^ T cells have been detected in humans, the overall contribution of HLA-E to the immune response to Mtb remains elusive, particularly whether it participates in inhibitory and/or immunoregulatory functions. In addition, whether murine Qa-1 can present Mtb peptides and its role in Mtb infection have not been investigated. In this study, we seek to extend and mechanistically dissect the findings from the human HLA-E studies to well controlled mouse TB models to better understand the diverse immunological functions of Qa-1/HLA-E in Mtb infection. We found that Qa-1 could present multiple Mtb peptide antigens to CD8^+^ T cells during aerosol Mtb infection, and Qa-1^-/-^ mice were more susceptible to Mtb infection compared to wild-type controls. CD8^+^ and CD4^+^ T cells in Mtb-infected Qa-1^-/-^ mice had more activated phenotypes and produced higher levels of pro-inflammatory cytokines, which have recently been associated with poor disease outcomes [43, 44]. In addition, CD8^+^ and CD4^+^ T cells in Mtb-infected Qa-1^-/-^ mice had increased expression of inhibitory and apoptosis-associated cell surface markers, correlated with a decreased ability to control bacteria. Together, our data suggest that Qa-1 participates in immune responses against Mtb through antigen-presentation and regulation of immune responses.

## Results

### Qa-1 expression is upregulated during aerosol *Mycobacterium tuberculosis* (Mtb) infection

**Fig 1:**
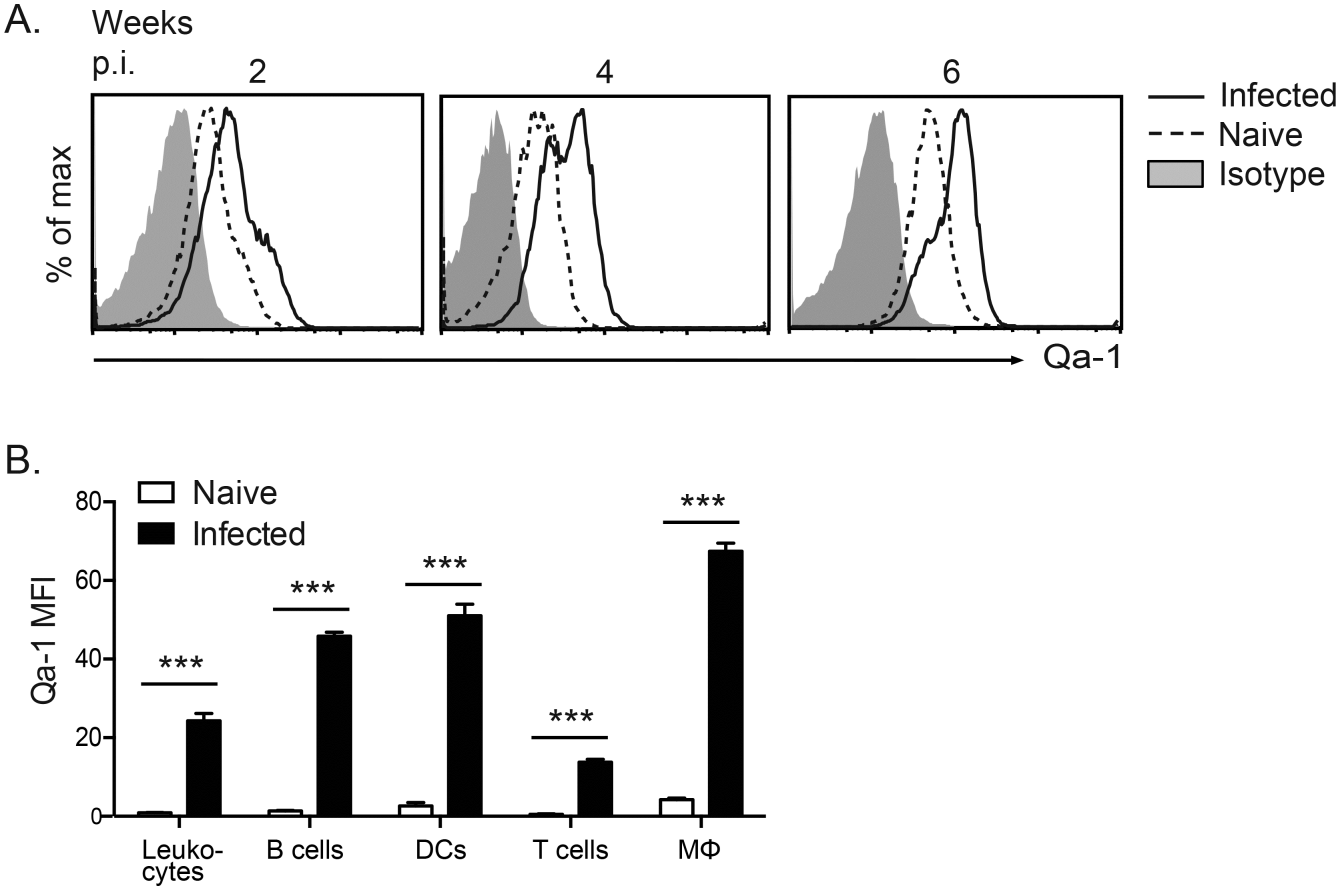
Qa-1 expression is upregulated during aerosol Mtb infection. (A) Expression of Qa-1 on leukocytes from mediastinal lymph node (MLN) of low-dose Mtb-infected C57BL/6 mice at 2, 4, and 6 weeks post-infection, as compared to uninfected B6 mice or isotype control. (B) MFI of Qa-1 expression on B cells (B220^+^ CD11c^-^), dendritic cells (DC) (CD11c^+^), T cells (TCRβ^+^), and macrophages (Mϕ) (CD11b^+^ F4/80^+^) from MLN of high-dose-infected B6 mice at 4 weeks post-infection, as compared to uninfected mice. Representative of 3 independent experiments, n ≥ 2 per group. * p < 0.05, ** p < 0.01, *** p < 0.001.

To determine if Qa-1 plays a role during Mtb infection, we first examined the surface expression of Qa-1. Low-dose aerosol Mtb infection with 100–200 CFU/lung is the most commonly used aerosol Mtb infection model, as it closely mimics the natural route of infection and disease progression. A high-dose aerosol Mtb infection model (1000–2000 CFU/lung) is also occasionally used to magnify phenotypic differences, particularly for mortality experiments [45]. We infected C57BL/6 (B6) mice with either a low- or high-dose of virulent Mtb strain H37Rv and found that Qa-1 expression was upregulated in both types of infection. In low-dose Mtb-infected B6 mice, increased expression of Qa-1 was detected on leukocytes from the mediastinal lymph nodes at 2 weeks post-infection, which continued to increase over the course of the infection (week 4 and 6, Fig 1A). Qa-1 expression in high-dose Mtb-infected mice was also increased relative to naïve mice, and was upregulated on multiple cell types, including T cells, B cells and other antigen-presenting cells, such as macrophages and dendritic cells (Fig 1B). These data suggest that Qa-1 may play a role in the immune response to Mtb infection.

### Several HLA-E-binding Mtb-peptides can also bind to Qa-1

**Fig 2:**
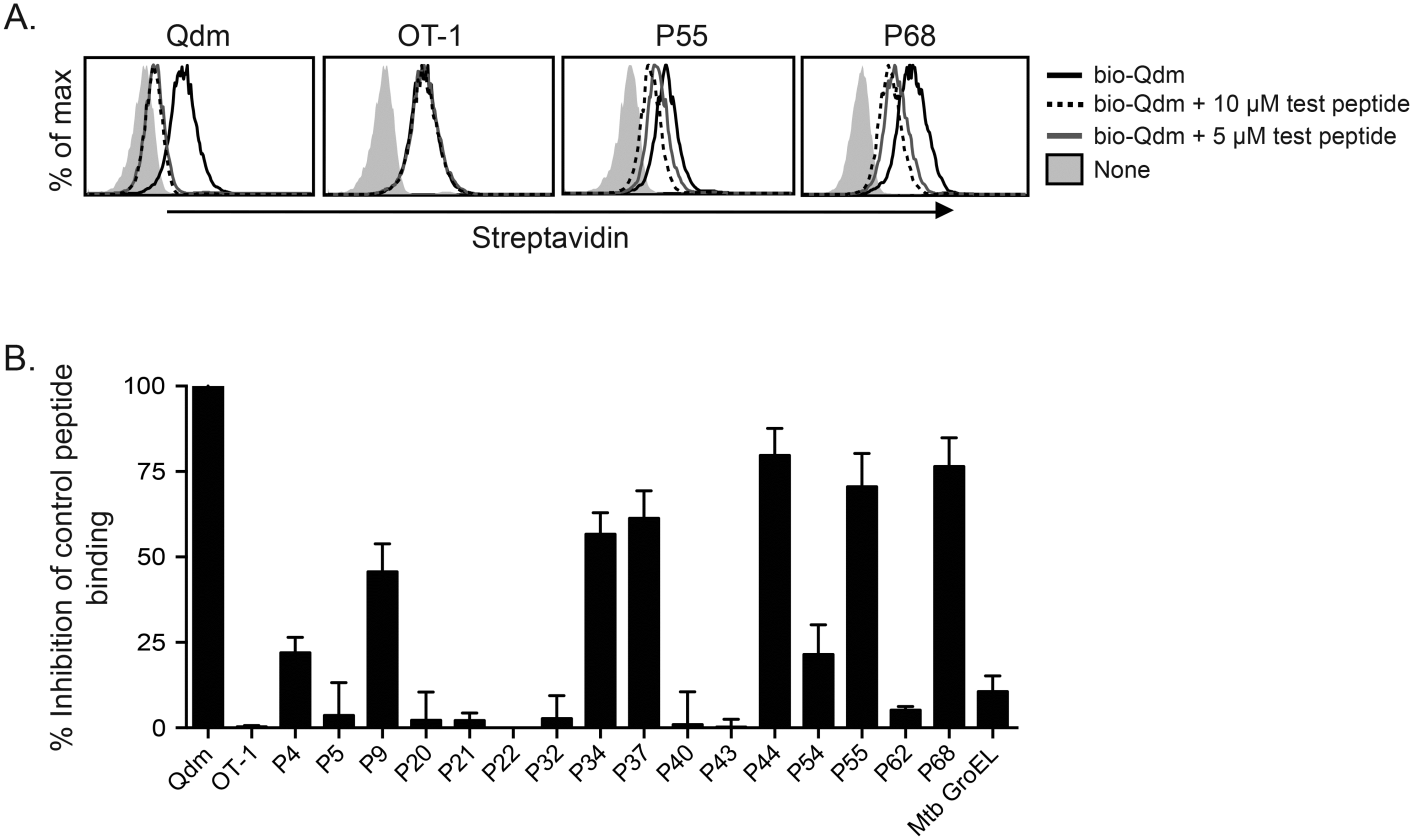
Several HLA-E-binding Mtb peptides can bind to Qa-1. Qa-1 transfected HeLa cells were incubated with 0.5 μM biotinylated-Qdm and either 5 or 10 μM of unlabeled competing peptide. Competition for binding to Qa-1 was determined via flow cytometry of streptavidin-APC staining. (A) Representative histograms of concentration-dependent peptide binding to Qa-1 for Qdm (positive control), OT-1 (negative control), and Mtb peptides P55 & P68. (B) Relative peptide binding for 17 Mtb peptides, determined by taking the difference in streptavidin-APC MFI between bio-Qdm alone and bio-Qdm + 10 μM test peptide, normalized to the inhibition of bio-Qdm binding by Qdm. n = 2–4 for each peptide.

Studies have shown that HLA-E can present Mtb peptides to CD8^+^ T cells in human TB [8, 18, 19]. As the antigen-binding grooves of Qa-1 and HLA-E share a high degree of structural similarity, we hypothesized that some HLA-E-binding peptides could also bind to Qa-1 and elicit Qa-1-restricted CD8^+^ T cell responses during Mtb infection. To test this hypothesis, a panel of Mtb peptides were synthesized (S1 Table) and tested for binding to Qa-1 in a flow cytometry-based peptide competition assay. The panel consisted of 17 peptides; of these, 16 were known to bind to HLA-E [8]. As the *Salmonella* GroEL chaperone protein has been shown to bind to Qa-1 [23], we also tested the Mtb GroEL homologue for binding to Qa-1. Qa-1-transfected HeLa cells were incubated with biotinylated Qdm (bio-Qdm) peptide and increasing concentrations of unbiotinylated competing test peptide. Peptide binding was determined by the subsequent decrease in streptavidin-APC staining intensity on HeLa-Qa1 transfectants due to displacement of bio-Qdm by competing peptide. While unlabeled Qdm peptide was able to bind to Qa-1 and displace bio-Qdm peptide, the negative control OT-I peptide was unable to do so (Fig 2A). Two representative Mtb peptides, P55 and P68, showed dose-dependent competition with bio-Qdm peptide (Fig 2A). Using this assay, we found six Mtb peptides that showed relatively high binding affinity to Qa-1 and were used for further study: P9, P34, P37, P44, P55, and P68 (Fig 2B).

### Qa-1 presents Mtb peptides to CD8^+^ T effector cells during aerosol Mtb infection

**Fig 3:**
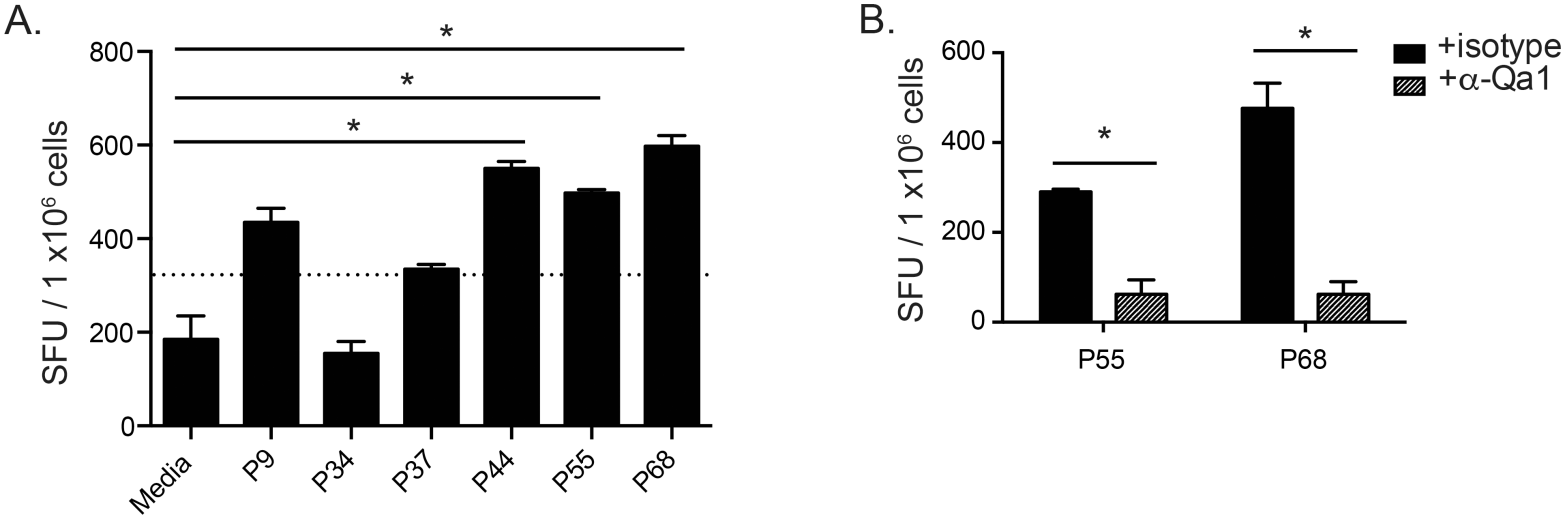
Qa-1 presents Mtb peptides to CD8^+^ T effector cells during aerosol Mtb infection. K^b-/-^D^b-/-^ mice were infected with a low-dose of Mtb, with splenic lymphocytes harvested at 4–6 weeks p.i. and enriched for CD8^+^ T cells. ELISpot assays were performed using enriched CD8^+^ T cells as responders and MHC II^-/-^ BMDC incubated with peptide or media as antigen presenting cells. (A) ELISpot of Mtb peptide-specific IFN-γ production from CD8^+^ T effector cells. Dotted line indicates 2 standard deviations above the mean of media alone control. Data representative of 3 independent experiments, n ≥ 4 mice each. (B) ELISpots as performed in (A), with either isotype or anti-Qa-1 antibody blocking. Data representative of 3 independent experiments, n ≥ 4 mice each. * p < 0.05.

To assess whether these Qa-1-binding Mtb peptides are readily presented to T cells and involved in the immune response during Mtb infection, we performed a low-dose Mtb infection on K^b-/-^D^b-/-^ mice. K^b-/-^D^b-/-^ mice were chosen because they lack MHC Ia molecules and may have a higher precursor frequency of Qa-1-restricted CD8^+^ T cells. At 4 weeks post-infection, CD8^+^ T cells were purified from the spleen and stimulated *in vitro* with bone marrow-derived dendritic cells (BMDC) presenting individual Mtb peptides. IFN-γ-producing Mtb peptide-specific CD8^+^ T cells were quantified by ELISPOT assays. Of the 6 Mtb peptides tested, CD8^+^ T cells specific to peptides P55 and P68 were the most frequently and consistently detected while the response to peptide P44 was rather variable between experiments (Fig 3A). The P55- and P68-specific IFN-γ responses could be blocked by the addition of a neutralizing anti-Qa-1 antibody (Fig 3B), confirming that peptides P55 and P68 are presented by Qa-1 to CD8^+^ T effector cells during Mtb infection, resulting in the production of IFN-γ. These P55 and P68-peptide-specific CD8^+^ T cells can also be detected in Mtb-infected B6 mice (S1A Fig). However, the magnitude of the response was lower than that found in K^b-/-^D^b-/-^ mice, and there was substantial variation in response between mice, likely due to a lower precursor frequency of Qa-1 restricted CD8^+^ T cells in B6 mice compared to K^b-/-^D^b-/-^ mice. Further, co-culture of CD8^+^ T cells from infected B6 mice with BMDC presenting P55 or P68 lead to an increase in the frequency of Annexin V expression on BMDCs relative to no peptide controls (S1B Fig). This peptide-specific increase in BMDC apoptosis was also dependent on the expression of Qa-1 by BMDCs. Together, these data suggest that Mtb peptide-specific Qa-1-restricted CD8^+^ T effector cells are present in Mtb-infected mice and may be able to induce peptide-specific cytotoxicity.

### Qa-1 deficiency results in increased bacterial burden and mortality in high-dose Mtb infection

**Fig 4:**
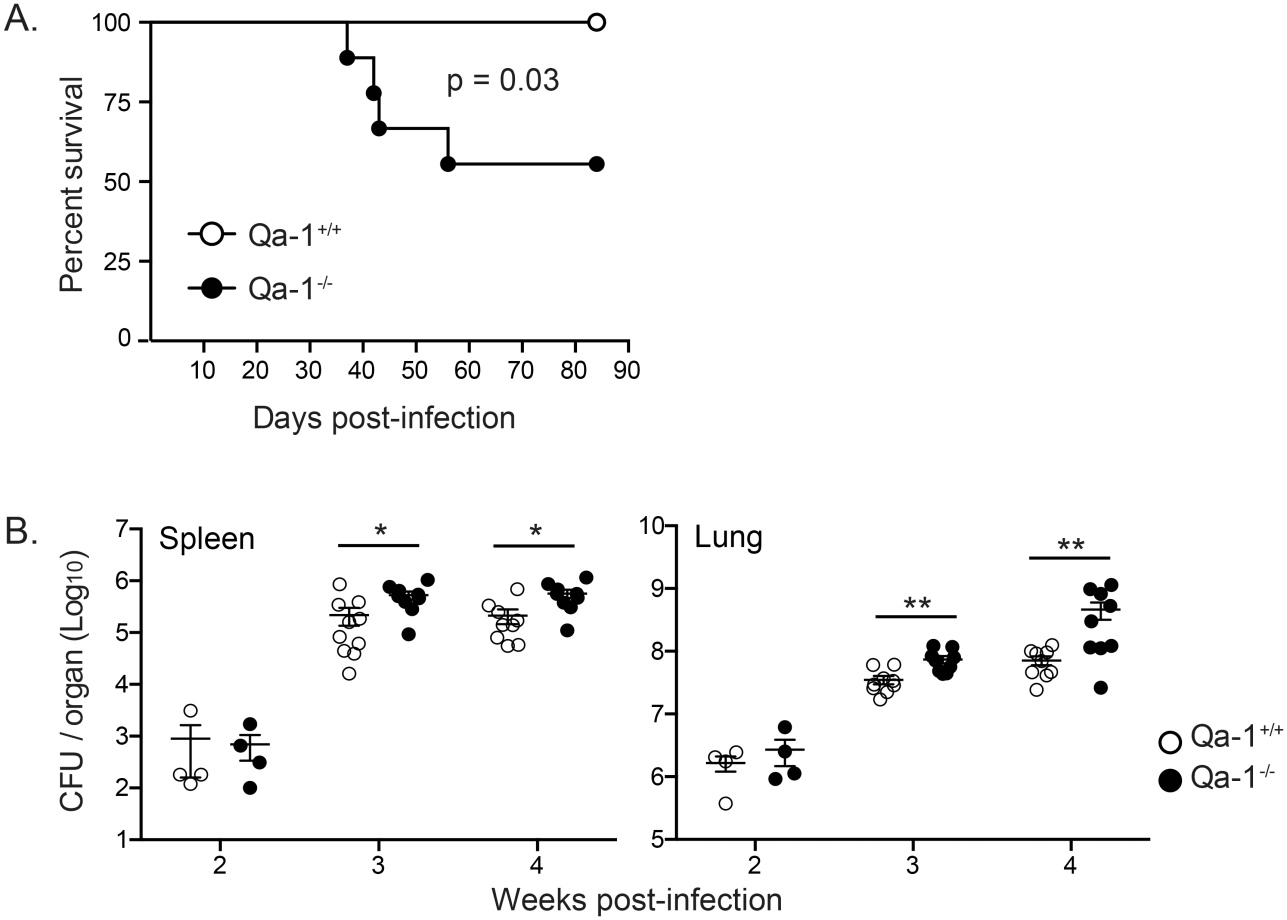
Qa-1 deficiency results in increased bacterial burden and mortality in high-dose Mtb infection. Age-matched, sex-matched Qa-1^+/+^ and Qa-1^-/-^ littermates were infected with a high-dose of aerosolized Mtb. (A) Survival time course for Qa-1^+/+^ and Qa-1^-/-^ mice after high-dose Mtb infection. n = 9 per group, representative of 3 independent experiments. Survival curve comparison p-value calculated with log-rank test. (B) Spleens and lung were harvested at indicated time points, homogenized, and plated for bacterial burden determination. Data at 2 weeks p.i. n = 4 per group, representative of 2 independent experiments. Data at 3 and 4 weeks p.i. pooled from 2 experiments, n ≥ 9 mice per group per time point. Data representative of at least 4 independent experiments. * p < 0.05, ** p < 0.01.

In addition to antigen presentation, Qa-1 has also been demonstrated to have immunoregulatory functions in viral infection and autoimmune disease models [35–37]. To determine what role Qa-1 plays in Mtb infection, we infected age-matched, sex-matched Qa-1^+/+^ and Qa-1^-/-^ littermates with either low- or high-dose aerosolized Mtb. We found that high-dose Mtb-infected Qa-1^-/-^ mice have a higher mortality rate compared to Qa-1^+/+^ mice. Qa-1^-/-^ mice began to succumb to high-dose Mtb infection shortly after 4 weeks post-infection, and showed close to 50% mortality by 90 days post-infection (Fig 4A). In contrast, no deaths in Qa-1^+/+^ littermates were found in this timeframe. In addition, high-dose Mtb-infected Qa-1^-/-^ mice had an increased bacterial burden in both the spleen and lung compared to Qa-1^+/+^ mice, beginning at 3 weeks post-infection (Fig 4B). These data indicate that Qa-1 is necessary for protection against high-dose aerosol Mtb infection. In contrast, there were no significant differences in bacterial burden between low-dose Mtb-infected Qa-1^+/+^ and Qa-1^-/-^ mice from 2 weeks up to 12 weeks post-infection (S2 Fig). Due to the significant phenotypes in bacterial burden and mortality, all later experiments were performed using high-dose Mtb infection.

### CD4^+^ and CD8^+^ T cells in Mtb-infected Qa-1^-/-^ mice produce higher levels of IFN-γ and have a more activated phenotype compared to their counterparts in Qa-1^+/+^ mice

**Fig 5:**
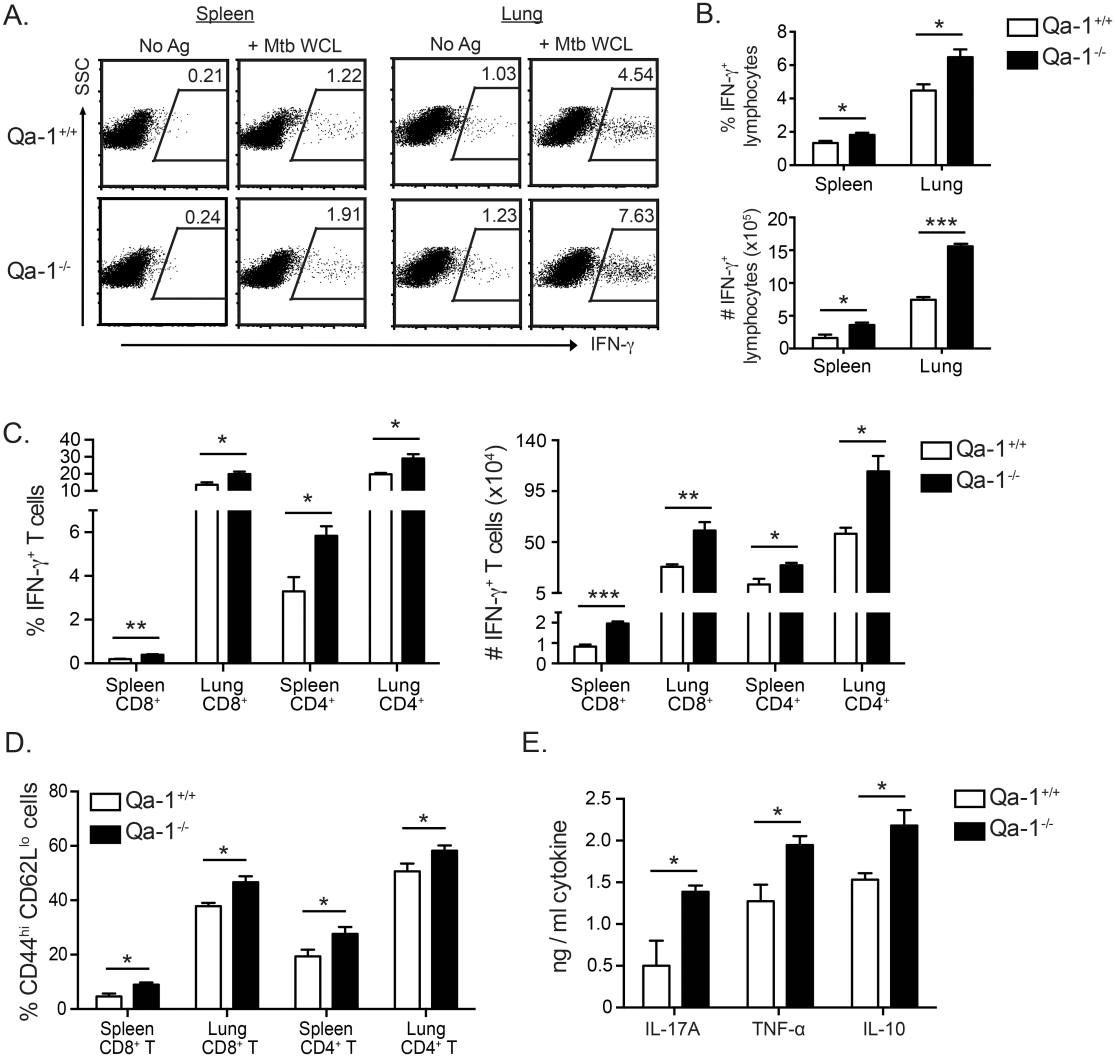
T cells from infected Qa-1^-/-^ mice produce higher levels of IFN-γ and have a more activated phenotype compared to Qa-1^+/+^ littermates. (A) Lymphocytes from indicated organs were isolated at 4 weeks p.i. and stimulated *ex vivo* with unpulsed (No Ag) or Mtb whole cell lysate-pulsed (+Mtb WCL) BMDC for 18h, then harvested for intracellular cytokine staining of IFN-γ. Qa-1^+/+^ lymphocytes were stimulated with Qa-1^+/+^ BMDC and Qa-1^-/-^ lymphocytes with Qa-1^-/-^ BMDC. Representative dot plots of Mtb antigen-specific intracellular IFN-γ expression. (B-C) IFN-γ intracellular cytokine staining was performed as described in (A), with frequency and total number of IFN-γ-expressing cells gated on total lymphocytes (B) or CD8^+^ and CD4^+^ T cells (C), from indicated organs. (D) Frequency of CD44^hi^ CD62L^lo^ CD8^+^ or CD4^+^ T cells in Qa-1^+/+^ and Qa-1^-/-^ mice at 4 weeks p.i., from indicated organs. (E) Lung lymphocytes from infected Qa-1^+/+^ and Qa-1^-/-^ mice were re-stimulated *in vitro* with Mtb WCL at 4 weeks p.i, with supernatant collected after 48 hours. Mtb antigen-specific cytokine production determined using CBA. (A-E) Data representative of 3 independent experiments, n≥4 mice per group. * p < 0.05, ** p < 0.01, *** p < 0.001.

To investigate how Qa-1 provides protection against Mtb infection, we began by analyzing the total cell number and function of T cells in Mtb-infected Qa-1^+/+^ and Qa-1^-/-^ mice. Flow cytometric analysis revealed no significant differences in the absolute number of CD8^+^ T cells, CD4^+^ T cells, or other types of leukocytes in the lung of Mtb-infected Qa-1^+/+^ and Qa-1^-/-^ mice (S3 Fig). Next, we analyzed antigen-specific IFN-γ production in Mtb-infected Qa-1^-/-^ and Qa-1^+/+^ mice using intracellular cytokine staining. Despite having increased bacterial burden and mortality, we found that Qa-1^-/-^ mice had a higher frequency and total number of Mtb antigen-specific, IFN-γ-producing lymphocytes compared to Qa-1^+/+^ mice (Fig 5A and 5B) in both the spleen and lung. Further, both CD4^+^ and CD8^+^ T cells in Mtb-infected Qa-1^-/-^ mice contributed to this increased production of IFN-γ (Fig 5C). Consistent with the enhanced IFN-γ production by T cells in Mtb-infected Qa-1^-/-^ mice, we found that these mice also had increased frequencies of activated CD44^hi^ CD62L^lo^ T effector cells (Fig 5D).

Besides IFN-γ, lymphocytes isolated from the lung of Mtb-infected Qa-1^-/-^ mice had increased production of pro-inflammatory IL-17A and TNF-α, as well as IL-10, in response to *in vitro* re-stimulation with Mtb whole cell lysate compared to Qa-1^+/+^ mice (Fig 5E). Neither Qa-1^-/-^ nor Qa-1^+/+^ mice produced significant amounts of IL-4 and IL-13 during Mtb infection. Both IFN-γ and TNF-α are critical cytokines in anti-Mtb immunity; both activate macrophages to kill intracellular bacteria, with TNF-α also participating in regulating granuloma formation and structure [2]. However, over-production of IFN-γ has recently been shown to be detrimental to the control of Mtb in the lung [43]. Although the role of IL-17A in Mtb infection is not well studied, it appears to play an important role in the generation of protective immune responses in the lung during early infection [46]. Despite increased pro-inflammatory cytokine production and more highly activated T cells, the Qa-1^-/-^ mice were unable to control Mtb infection as well as Qa-1^+/+^ mice, indicating that Qa-1 deficiency results in over-activation of the T cell response to Mtb infection.

### CD4^+^ and CD8^+^ T cells in Mtb-infected Qa-1^-/-^ mice have an increased expression of inhibitory markers

**Fig 6:**
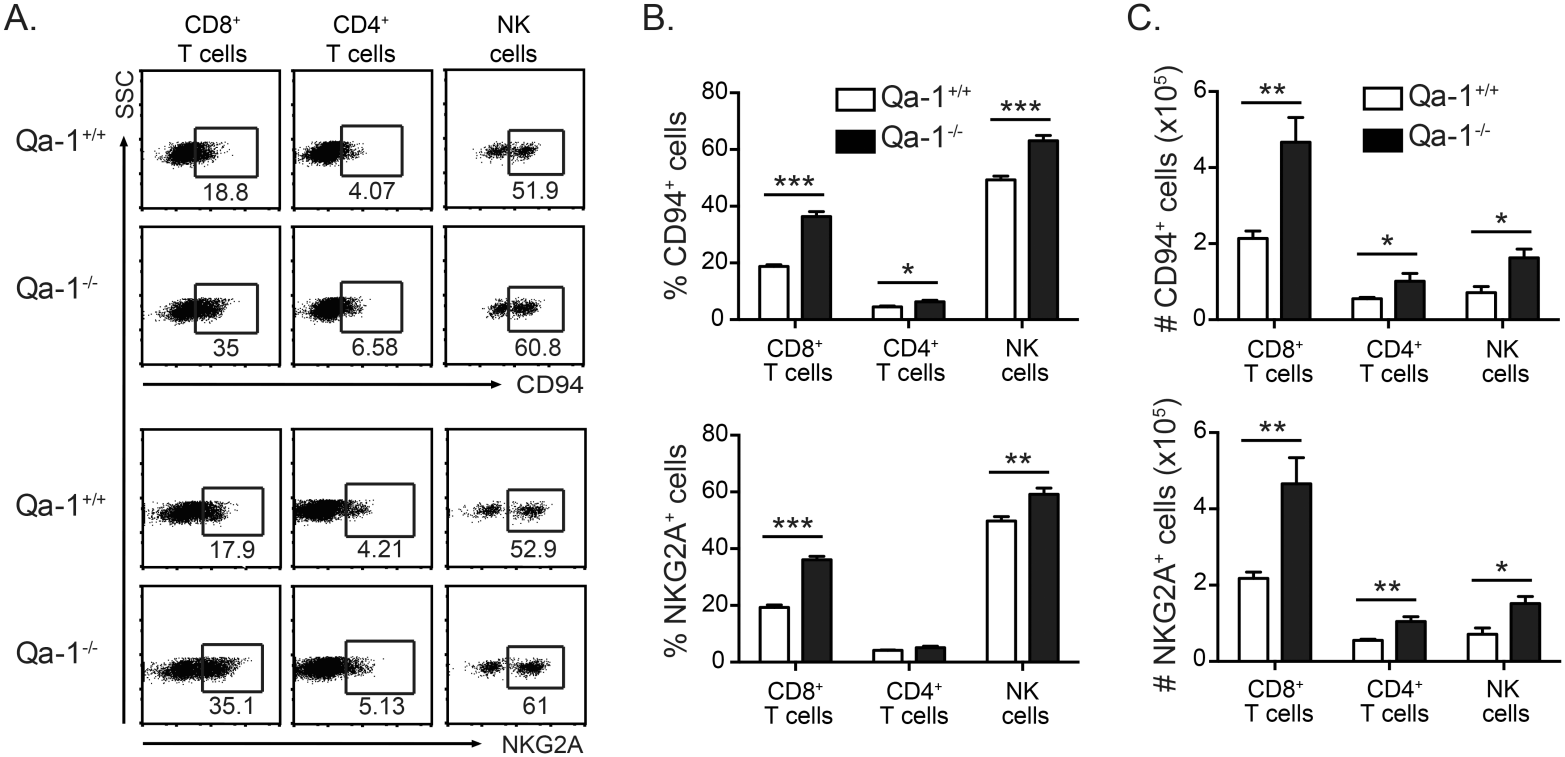
T cells from infected Qa-1^-/-^ mice have increased expression of inhibitory NK receptors. Lung lymphocytes from Qa-1^**+/+**^ and Qa-1^**-/-**^ mice were harvested at 4 weeks p.i. and surface expression of indicated markers analyzed by flow cytometry. (A) Representative dot plots of CD94 and NKG2A expression by cell type. (B-C) Frequency and total number of CD94- and NKG2A-expressing CD8^+^ T cells, CD4^+^ T cells, and NK cells. Data representative of at least 3 independent experiments, n≥4 mice per group. * p < 0.05, ** p < 0.01, *** p < 0.001.

We next explored the mechanisms by which Qa-1 can downregulate immune responses during Mtb infection. Inhibitory NK receptors CD94/NKG2A have been shown to be upregulated on a subset of activated CD8^+^ T cells and possibly CD4^+^ T cells in response to infection [28, 29]. Further, negative regulation of CD8^+^ T cell responses by CD94/NKG2A during poxvirus infection has been shown to prevent over-activation and subsequent apoptosis of CD8^+^ T cells, thereby enhancing the anti-viral immune response [36]. In naïve Qa-1^+/+^ and Qa-1^-/-^ mice, the expression of CD94 and NKG2A, as well as activating NK receptor Ly49D, was not significantly different (S4 Fig). During Mtb infection, we found that while lymphocytes from both Qa-1^+/+^ and Qa-1^-/-^ mice upregulated CD94 and NKG2A expression, Qa-1^-/-^ mice had significantly higher frequencies and absolute numbers of NK cells and T cells (especially CD8^+^ T cells) that expressed inhibitory CD94/NKG2A receptors compared to Qa-1^+/+^ mice (Fig 6A–6C). Other members of the NKG2 family, such as NKG2C and NKG2E, also dimerize with CD94 to form activating receptors. As there is no antibody specifically for NKG2C or NKG2E, it was necessary to use the 20d5 antibody clone to detect NKG2A/C/E. Expression of NKG2A/C/E was virtually identical to that of NKG2A, indicating inhibitory NKG2A is the dominant form expressed in Mtb-infected mice, with very low expression of activating receptors NKG2C and NKG2E (S5A Fig). In addition, we performed quantitative PCR and confirmed the low expression of NKG2C/E relative to NKG2A on T cells from Mtb-infected mice (S5B Fig). The increased expression of CD94/NKG2A inhibitory receptor along with the increased production of IL-10 regulatory cytokine (Fig 5E) in Mtb-infected Qa-1^-/-^ mice indicate that Qa-1-deficient mice have dysregulated immune responses during Mtb infection.

As the expression of inhibitory CD94/NKG2A receptor was also increased on NK cells in Mtb-infected Qa-1^-/-^ mice, we examined if there were differences in NK cell function between Qa-1^+/+^ and Qa-1^-/-^ mice. We used a high dose, intravenous Mtb infection model known to stimulate NK cell function [47] and examined the cytotoxic potential and IFN-γ production of NK cells in Qa-1^+/+^ and Qa-1^-/-^ mice. Qa-1^-/-^ mice did not show any differences in the number of IFN-γ-producing NK cells or lysis of YAC-1 target cells compared to Qa-1^+/+^ mice (S6 Fig).

### T cells in Mtb-infected Qa-1^-/-^ mice display increased apoptosis and decreased ability to mediate killing of Mtb

**Fig 7:**
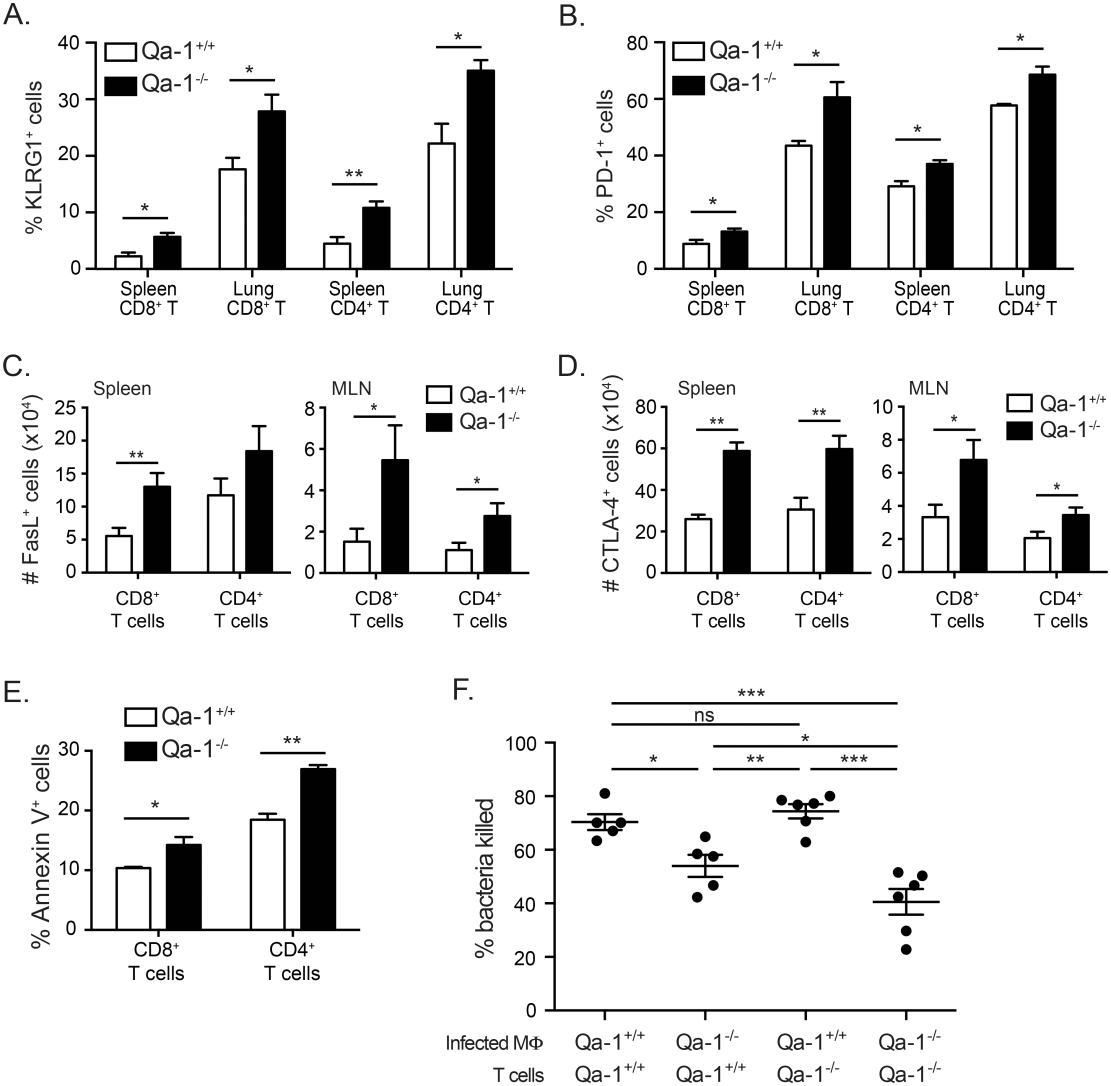
T cells in Mtb-infected Qa-1^-/-^ mice have increased apoptosis and decreased ability to promote killing of Mtb by macrophages. Lymphocytes from the lung, spleen, or mediastinal lymph node of Qa-1^**+/+**^ and Qa-1^**-/-**^ mice were harvested at 4 weeks p.i for surface marker expression flow cytometry (A-E) and functional assays (F). (A-B) Frequency of KLRG1 or PD-1 expression on CD8^+^ and CD4^+^ T cells from indicated sites of infection. n = 3–5 mice per group, representative of 3 independent experiments. (C-D) Total number of FasL^+^ or CTLA-4^+^ CD8^+^ or CD4^+^ T cells from indicated sites of infection. n = 6–8 mice per group, pooled from 2 experiments. (E) Frequency of Annexin V^+^ CD8^+^ or CD4^+^ T cells in mediastinal lymph node of infected mice. n = 3 mice per group, representative of 2 independent experiments. (F) Purified splenic T cells from infected Qa-1^+/+^ and Qa-1^-/-^ mice were co-cultured with Qa-1^+/+^ or Qa-1^-/-^ BMDM *in vitro* infected with Mtb. Co-cultures were incubated for 6 days and then plated for CFU determination. Bacterial killing shown relative to CFU count in infected BMDM alone. Data pooled from 2 experiments, representative of 4 independent experiments. * p < 0.05, ** p < 0.01, *** p < 0.001.

Further characterization of the surface phenotype of T cells in Mtb-infected Qa-1^+/+^ and Qa-1^-/-^ mice showed that a high proportion of T cells in Qa-1^-/-^ mice expressed several other inhibitory and apoptotic markers. T cells in Mtb-infected Qa-1^-/-^ mice showed an increased frequency of KLRG1 and PD-1 expression (Fig 7A and 7B). KLRG1 has been shown to be expressed on terminally differentiated CD8^+^ and CD4^+^ T cells that are able to produce cytokines upon antigen stimulation but have poor proliferation in viral and Mtb infection models [48, 49]. PD-1 has been shown to downregulate immune responses through the induction of apoptosis and is often used to identify activated or exhausted T cells. Mtb-infected Qa-1^-/-^ mice also had a larger number of T cells expressing FasL and CTLA-4 compared to Qa-1^+/+^ mice (Fig 7C and 7D). FasL is both a marker for activated T cells as well as an important ligand for induction of activation induced cell death (AICD). Similarly, CTLA-4 is expressed on activated conventional T cells, but also provides an inhibitory signal for T cell function when bound by CD80 or CD86. In combination with the increased expression of inhibitory and apoptotic cell surface markers, both CD4^+^ and CD8^+^ T cells from Mtb-infected Qa-1^-/-^ mice had a higher frequency of Annexin V^+^ cells in the mediastinal lymph node, indicating increased cell death (Fig 7E). Lastly, we examined T cell-mediated macrophage killing of Mtb. Purified T cells from Mtb-infected Qa-1^+/+^ and Qa-1^-/-^ mice were co-cultured with either Qa-1^+/+^ or Qa-1^-/-^ bone marrow derived macrophages (BMDM) that were *in vitro* infected with Mtb. We found that T cells from Qa1^-/-^ mice cultured with Qa-1^-/-^ macrophages were the least effective in promoting BMDM to kill the intracellular Mtb (Fig 7F). While Qa-1^+/+^ T cells cultured with Qa-1^+/+^ BMDM resulted in efficient bacterial clearance, Qa-1^+/+^ T cells cultured with Qa-1^-/-^ BMDM were not as efficient at promoting bacterial killing. This indicates that the absence of Qa-1 expression on antigen presenting cells can cause dysregulation of immune function even in an *in vitro* setting. Taken together, our data demonstrates that aberrant activation of T cells from Qa-1^-/-^ mice in turn leads to increased T cell death and an inferior ability of these T cells to mediate control of bacterial burden, which is in line with our mortality and in vivo bacterial burden experiments (Fig 4).

### Both CD8^+^ T_regs_ and inhibitory CD94/NKG2A receptors are involved in Qa-1-mediated regulation of anti-Mtb immune responses

Qa-1 has been shown to modulate immune responses through two different mechanisms: the activation of Qa-1-restricted CD8^+^ T_reg_ cells, or the interaction of Qa-1/Qdm with inhibitory CD94/NKG2A receptors [24]. Thus, the dysregulated immune response in Mtb-infected Qa-1^-/-^ mice was either due to a lack of CD8^+^ T_reg_ suppression of T cell responses or a lack of Qa-1 binding to inhibitory CD94/NKG2A receptors on activated T cells. Unlike CD4^+^ T regulatory cells, CD8^+^ T_regs_ cannot be defined by any one set of cell surface markers or transcription factors. Recent studies from TB patient samples have identified suppressive CD8^+^ T cells expressing CD25 and FoxP3 [50, 51], among other markers. In addition, Qa-1-restricted CD8^+^ T_regs_ have been found to be enriched among CD44^hi^ CD122^+^ Ly49^+^ CD8^+^ T cells in primarily autoimmune disease settings [52, 53]. We used the markers from these human and mouse studies to potentially identify CD8^+^ T_regs_ in Mtb-infected mice. However, we found no differences in the number of CD25^+^ FoxP3^+^ CD8^+^ T cells or CD44^+^ CD122^+^ Ly49^+^ CD8^+^ T cells in infected Qa-1^+/+^ compared to Qa-1^-/-^ mice (S7 Fig), indicating that these markers may not be applicable in identifying Qa-1-restricted CD8^+^ T_regs_ in Mtb infection. To determine which mechanism was responsible for Qa-1-mediated immune regulation during Mtb infection, we instead turned to genetically modified Qa-1 mutant knock-in mice: Qa-1 R72A and Qa-1 D227K. The Qa-1 R72A mutation disrupts binding to NKG2A receptors, removing the inhibitory interaction between Qa-1/Qdm and CD94/NKG2A receptors but maintaining the ability to activate CD8^+^ T_regs_ [54]. The D227K mutation disrupts the interaction of Qa-1 with the CD8 co-receptor, resulting in mice that have no Qa-1-restricted CD8^+^ T_regs_, but do have a functional interaction between Qa-1/Qdm and CD94/NKG2A [55].

**Fig 8:**
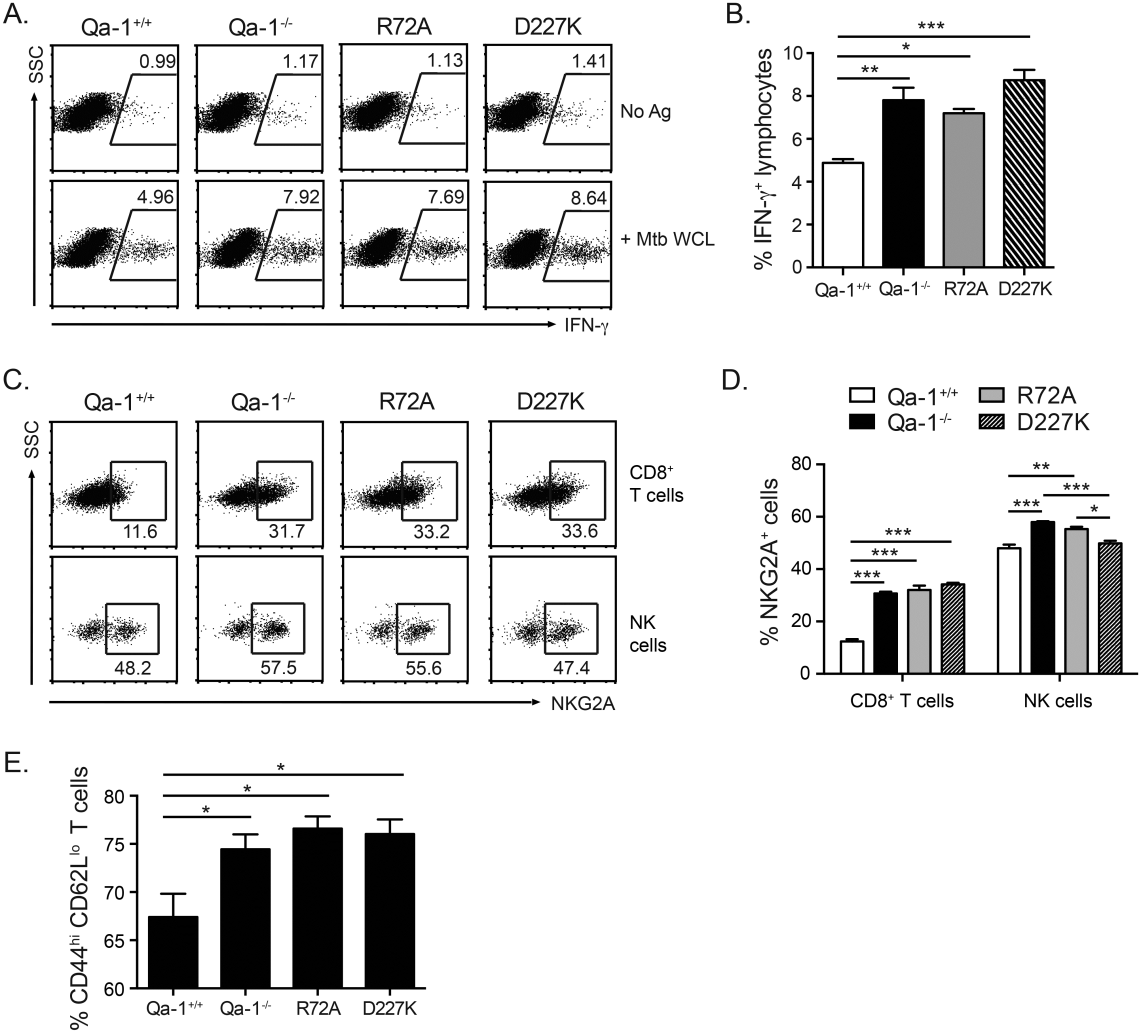
T cells in Qa-1 R72A and D227K mice are phenotypically similar to Qa-1^-/-^ mice. Lung lymphocytes from Mtb-infected Qa-1^**+/+**^, Qa-1^**-/-**^, Qa-1 R72A, and Qa-1 D227K mice were harvested at 4 weeks p.i and analyzed by flow cytometry. (A-B) Lung lymphocytes were stimulated *ex vivo* with unpulsed (No Ag) or Mtb whole cell lysate-pulsed (+Mtb WCL) BMDC for 18h, then harvested for intracellular cytokine staining. (A) Representative dot plots of Mtb antigen-specific intracellular IFN-γ expression. (B) Frequency of IFN-γ expressing lymphocytes from indicated genotypes. (C) Representative dot plots and (D) frequency of NKG2A-expressing CD8^+^ T and NK cells from infected mice. (E) Frequency of CD44^hi^ CD62L^lo^ T cells from infected mice. Data representative of at least 3 independent experiments, n≥4 mice per group. * p < 0.05, ** p < 0.01, *** p < 0.001.

Analysis of Mtb-infected Qa-1^+/+^, Qa-1^-/-^, Qa-1 R72A, and Qa-1 D227K mice showed that Qa-1 R72A and D227K mutants have an increased frequency of IFN-γ-producing cells compared to Qa-1^+/+^ mice (Fig 8A and 8B). In fact, no differences in the frequency of IFN-γ- producing cells were found among Qa-1^-/-^, R72A, and D227K mice. In addition, the frequency of NKG2A expression was increased on CD8^+^ T cells from Qa-1^-/-^, R72A, and D227K mice, as compared to Qa-1^+/+^ mice (Fig 8C and 8D). Lastly, the frequency of activated CD44^hi^ CD62L^lo^ T effector cells was also increased in Qa-1^-/-^, R72A, and D227K mice as compared to Qa-1^+/+^ mice (Fig 8E). These data show that T cells from both Qa-1 knock-in mutants had a phenotype similar to that of Qa-1^-/-^ mice, indicating that both CD8^+^ T_reg_ cells and inhibitory CD94/NKG2A receptors are involved in regulating immune responses during Mtb infection. Interestingly, NKG2A expression on NK cells in Qa-1 D227K mice was comparable to that of Qa-1^+/+^ NK cells, but significantly decreased from that of Qa-1^-/-^ and R72A (Fig 8C and 8D). The differential regulation of NKG2A expression on CD8^+^ T cells and NK cells observed in Qa-1 R72A and Qa-1 D227K mice suggests that the interaction of Qa-1, NKG2A and CD8/TCR play a direct role in regulating NKG2A expression during Mtb infection.

## Discussion

Qa-1 has been implicated in the immune response in many different disease models, including intracellular bacterial infections, viral infections, and autoimmune diseases. Depending on the disease model, Qa-1 can function as an antigen presentation or immune regulation molecule. Similar to HLA-E, we found that Qa-1 can bind and present Mtb peptide antigens to CD8^+^ T cells during aerosol Mtb infection, resulting peptide-specific IFN-γ production and cytotoxicity to antigen presenting cells. In addition, our study demonstrates that Qa-1 provides protection against Mtb infection by restraining T cell immune responses, preventing T cell over-activation and death, leading to more effective bacterial clearance.

Nonclassical MHC Ib molecules HLA-E and Qa-1 may be well suited to Mtb peptide vaccine development due to their low polymorphic character. In particular, HLA-E expression has been found to be enriched in phagosomes containing Mtb, which may facilitate loading of Mtb peptides onto HLA-E in infected cells [56]. Unlike MHC Ia molecules, HLA-E surface expression is not downregulated on HIV-infected human cells [57], likely as a pathogen response to evade NK cell activity. This adaptation could be exploited by HLA-E-restricted CD8^+^ T cells, considering the high incidence of TB and HIV coinfection. Our study detected Qa-1-restricted CD8^+^ T effector cells specific to Mtb peptides P55 and P68 from Mtb-infected mice. As these two peptides were also among the most consistently detected HLA-E-restricted responses in human studies [8, 49], our findings indicate they may be promising targets for future vaccination studies. We attempted to immunize naïve mice with P55 and P68 peptide-pulsed dendritic cells to increase the frequency of these Mtb-specific T cells, but were unable to generate CD8^+^ T cells with consistent peptide-specific IFN-γ production upon restimulation from immunized mice. One issue for peptide immunization may be that HLA-E- and Qa-1-peptide complexes are significantly less stable than MHC Ia-peptide complexes at physiologic temperature, with rapid turnover of Qa-1 surface expression even when bound to Qdm [58]. Improved alternative immunization methods, such as the use of modified viral vectors to preferentially induce HLA-E-restricted CD8^+^ T cell responses [59], may be needed to optimize both initial presentation of peptide and subsequent detection of peptide-specific responses.

Although the role of CD4^+^ T regulatory cells in the context of modulating immune responses to pathogens has been well studied, CD8^+^ T_regs_ have not, particularly in Mtb infection. One reason for the relative lack of insight into CD8^+^ T_reg_ function during infectious disease is because unlike CD25^+^ FoxP3^+^ CD4^+^ T regulatory cells, there are no universal markers for the identification of CD8^+^ T_regs._ While a few studies have identified cell surface markers that enrich for CD8^+^ T_reg_ populations from TB patient samples based on suppressive activity [50, 51], it is unclear how specific these markers are across different disease models, particularly for mouse models of infection. The best-known subpopulation of suppressive CD8^+^ T cells are the mouse Qa-1-restricted CD8^+^ T_regs_. Qa-1-restricted CD8^+^ T_regs_ have primarily been studied in the context of autoimmune diseases such as EAE and systemic lupus erythematosus, where these CD8^+^ T_regs_ suppress autoreactive CD4^+^ T cells in an antigen-specific manner and protect against disease progression [24, 41]. Knowledge of the restriction element of this major subset of CD8^+^ T_regs_ allowed for genetic depletion of these suppressive cells without needing to specifically identify them by surface phenotype. We were thus able to use mice deficient in either Qa-1-restricted CD8^+^ T_regs_ (Qa-1 D227K mutant) or the inhibitory NKG2A interaction (Qa-1 R72A mutant) to tease apart the regulatory contributions for both. Our data showed that, similar to Qa1^-/-^ mice, T cells from both R72A and D227K mutants were more activated and had increased IFN-γ production compared to wild-type mice, suggesting both mechanisms are involved in regulating immune responses in Mtb infection. It is important to note that while Qa-1 D227K mutant mice are deficient in Qa-1-restricted CD8^+^ T_regs_, they may also have impaired Qa-1-restricted CD8^+^ T effector cells. Further experimentation is needed to determine the total contribution of these CD8^+^ T effector cells to the anti-mycobacterial immune response compared to suppressive CD8^+^ T cells.

Although our study is among the first to report participation of inhibitory NK receptors on T cells in the modulation of anti-Mtb immune responses [60, 61], inhibitory CD94/NKG2A has been implicated in a number of anti-viral T cell response studies. Blockade or deletion of NKG2A resulted in increased inflammatory cytokine production, inflammatory cell infiltrate at sites of infection, and tissue injury in influenza and adenovirus infection models [33, 34]. Further, CD94/NKG2A expression is regulated by TCR engagement and cytokines, with IL-6, IL-10, and IL-21 upregulating expression and IL-4, IL-23, and IL-2 downregulating expression [62]. However, whether the overall effect of NKG2A-mediated immunoregulation results in decreased T cell function or more efficient response against pathogens appears to be related to the particular model being studied. While NKG2A engagement restricted CD8^+^ T cell cytotoxic ability against polyoma virus, potentially leading to more virus-induced tumors [35], NKG2A prevented over-activation and apoptosis of poxvirus-specific CD8^+^ T cells, preserving their ability to respond to the pathogen [36]. In our model, the increased expression of inhibitory CD94 and NKG2A on T cells, combined with increased IL-10 production in Qa-1^-/-^ mice are signs of an aberrant immune response that needs to be downregulated. As Qa-1^-/-^ mice do not express the Qa-1/Qdm complex, however, they are unable to initiate the CD94/NKG2A regulatory signaling cascade and the T cells remain over-activated. Increased T cell activation has recently been shown to be an indicator of risk of TB progression in humans [44]. It is likely that the persistent over-activation of these T cells in our mouse model in turn leads to increased expression of other inhibitory and apoptosis-associated cell surface markers, increased cell death, and the inability of these T cells to control bacterial burden.

Using Qa-1-deficient mice, our study is the first to describe the overall contribution of the Qa-1 molecule to the immune response against Mtb. We found that while Qa-1 is able to present Mtb peptides in infected Qa-1-sufficient mice, Qa-1^-/-^ mice are less able to protect against Mtb infection compared to Qa-1^+/+^ littermates, due to aberrant activation and function of CD4^+^ and CD8^+^ T lymphocytes. Using genetically manipulated mouse models, we demonstrated that the Qa-1-mediated regulation of CD4^+^ and CD8^+^ T cells in Mtb infection is achieved through the interaction with inhibitory NK receptors on activated T cells and the presence of Qa-1-restricted CD8^+^ T_reg_ cells. Given Qa-1’s antigen presentation and regulatory roles, it is possible that Mtb-specific Qa-1-restricted CD8^+^ T effector cells could have both cytolytic and regulatory functions. Indeed, a number of Mtb-specific HLA-E-restricted CD8^+^ T cell clones and polyclonal CD8^+^ T cells showed both cytolytic and suppressive capacity [8, 49]. In the future, it would be of interest to determine how CD8^+^ T_regs_ and activating/inhibitory NK receptors participate in the human anti-mycobacterial immune response. We anticipate that our study will inform these future murine and human Qa-1/HLA-E studies, leading to a better understanding of protective immune responses against Mtb.

## Materials and methods

### Ethics statement

This study was carried out in strict accordance with the recommendations in the Guide for the Care and Use of Laboratory Animals of the National Institutes of Health. The protocol was approved by the Animal Care and Use Committee of the Northwestern University (Protocol number: IS00000985).

### Mice

K^b-/-^D^b-/-^ on the B6 background were provided by Dr. James Forman (UT Southwestern Medical Center, Dallas, TX) and maintained in house. Qa-1^-/-^, Qa-1 R72A, and Qa-1 D227K mutant mice mice were provided by Dr. Harvey Cantor (Dana-Farber Cancer Institute, Boston, MA). Qa-1^-/-^ mice were crossed with C57BL/6 from Jackson Laboratories (Bar Harbor, ME) to generate Qa-1^+/-^, Qa-1^-/-^, and Qa-1^+/+^ littermates [41]. All mice were housed in a specific pathogen free environment at Northwestern University.

### Mycobacterial antigens

Mtb H37Rv whole cell lysate was obtained through BEI Resources. Mtb peptides were synthesized by Peptide 2.0 (Chantilly, VA). Mtb antigens were dissolved in either DMSO or PBS and stored as aliquots at -20°C.

### Qa-1/peptide binding assay

Qa-1 transfected HeLa cells were a gift from Dr. James Forman (UT Southwestern Medical Center, Dallas, TX) were incubated with 0.5 μM biotinylated Qdm (bio-Qdm) and either 5 μM or 10 μM of unbiotinylated competing Mtb or control peptide for 1.5 hours at 4°C. Cells were then washed with HBSS/2% FBS, stained with streptavidin-APC (BioLegend, San Diego, CA), and flow cytometry was performed to determine the inhibition of bio-Qdm binding by competing peptide.

### Aerosol *Mycobacterium tuberculosis* infection

Frozen aliquots of Mtb H37Rv were thawed and diluted in PBS with 0.05% Tween 80. Mice were infected with either low-dose (100–200 CFU) or high-dose (~1000 CFU) of Mtb using a nose-only aerosol exposure chamber (In-Tox Products, NM), equipped with a miniHEART nebulizer (WestMed, Tucson, AZ), as previously described [11]. A day 1 CFU count was performed to determine the infecting dose. At indicated time-points after infection, bacterial loads in the lungs and spleens were determined by plating serial dilutions of tissue homogenate on Middlebrook 7H11 agar plates (BD, Franklin Lakes, NJ), and colonies were counted after 2–3 weeks of incubation at 37°C.

### Lymphocyte isolation and dendritic cell generation

Single-cell suspensions were prepared from the lung, spleen and mediastinal lymph nodes by mechanical disruption in HBSS/2% FBS, followed by culture in complete RPMI media. Lung was digested with collagenase IV (1 mg/ml) and DNase I (30 μg/ml) (Sigma, St. Louis, MO) for 30 min. at 37°C prior to mechanical disruption. For ELISpot assays, CD8^+^ T cells were enriched using negative selection with biotinylated mAb specific to CD4 (GK1.5), CD11b (M1/70), and B220 (RA36B2) (BioLegend, San Diego, CA) followed by streptavidin-conjugated magnetic beads (Invitrogen, Carlsbad, CA). For RNA extraction, CD8^+^ and CD4^+^ T cells were isolated using positive selection with biotinylated mAbs specific to CD8β (YTS156.7.7), followed by streptavidin-conjugated magnetic beads (Miltenyi Biotech, San Diego, CA) or CD4 Microbeads directly (Miltenyi Biotech, San Diego, CA), followed by purification via magnetic column (Miltenyi Biotech). The purity and composition of enriched T cells were confirmed by flow cytometry. Bone marrow-derived dendritic cells (BMDCs) were derived from mouse bone marrow progenitors using GM-CSF and IL-4 (PeproTech, Rocky Hill, NJ) as previously described [11].

### ELISpot assay

IFN-γ ELISpot assay was performed as previously described [11], with some modifications. Briefly, multiscreen-IP plates (Millipore, Bedford, MA) were coated with anti-IFN-γ mAb (AN-18, BioLegend, San Diego, CA) at 5μg/ml in PBS. Enriched CD8^+^ T cells from infected mice were incubated with MHC II^-/-^ BMDCs with media alone or 5μM of Mtb peptide, in duplicate. To confirm Qa-1 restriction, BMDCs were pre-incubated with 2 μg/ml anti-Qa-1 blocking mAb (6A8.6F10.1A6, BD, Franklin Lakes, NJ) or mouse IgG1 isotype control (clone MOPC-21) (BioXCell, West Lebanon, NH) prior to adding peptide and lymphocytes. After 18h incubation at 37°C, plates were washed using PBS/0.05% Tween 20 and developed using biotinylated α-IFN-γ mAb (R4.6A2, eBioscience, San Diego, CA), followed by streptavidin-conjugated alkaline phosphatase (Jackson ImmunoResearch Laboratories, West Grove, PA) and a BCIP/NBT substrate kit (Bio-Rad, Hercules, CA) according to the manufacturer’s instructions. Spots were counted using an ImmunoSpot reader (Cellular Technology, Shaker Heights, OH).

### Antibodies and flow cytometry

Cells were incubated with 2.4G2 Fcγ RII/RIII blocking mAb for 15 min, then stained with appropriate combinations of mAbs in HBSS/2% FBS for 30 min at 4°C. Monoclonal antibodies against mouse Qa-1 (6A8.6F10.1A6), TCRβ (H57-597), NK1.1 (PK136), CD4 (GK1.5), CD8β (YTS156.7.7), CD11c (N418), CD11b(M1/70), F4/80 (BM8), Ly6G(1A8), CD44 (1M7), CD62L (MEL14), PD-1 (29F.1A12), KLRG1 (2F1/KLRG1), B220 (RA36B2), CD94 (18d3), NKG2A (16A11), NKG2ACE (20d5), NKG2D (C7), Ly49D (4E5), FasL (MFL3), CTLA-4 (UC10-4B9), FOXP3 (150D), CD25(PC61), CD122(TM-β1), and Ly49C/F/I/H (14B11) with different fluorochrome conjugates were purchased either from BioLegend, eBioscience, or BD Bioscience (San Diego, CA). Transcription factor staining was performed using the FOXP3/Transcription Factor Staining Set (eBioscience, San Diego, CA) according to manufacturer’s directions. Annexin V staining was performed after lymphocytes were incubated for 5 hours in culture media at 37°C. Annexin V staining was done in Annexin V Binding Buffer (BioLegend, San Diego, CA) according to manufacturer’s directions. Flow cytometry was performed with a FACS CantoII (BD Biosciences, San Jose, CA) and analyzed using FlowJo software (Tree Star, Ashland, OR).

### Intracellular cytokine staining

Lymphocytes from the spleen and lung of infected mice were stimulated with unpulsed or Mtb whole cell lysate-pulsed (10 μg/mL) BMDCs. After two hours of incubation, 5μg/ml Brefeldin A (Sigma, St. Louis, MO) was added and cells were cultured for an additional 16 hours. Cells were then stained for cell surface markers, fixed with 4% paraformaldehyde, permeabilized with 0.2% saponin, and then stained with APC-conjugated α-IFN-γ (BioLegend, San Diego, CA). Flow cytometry was performed as described.

### Cytokine measurement using cytometric bead array

Lymphocytes from Mtb-infected mice were incubated with Mtb whole cell lysate (10 μg/mL) for 40 hours at 37°C. Supernatants were harvested and the cytokines IL-17A, IFN-γ, TNF-α, IL-4, IL-13 and IL-10 were detected using individual mouse cytokine Cytometric Bead Array (CBA) flex sets (BD, San Jose, CA), per manufacturer’s directions. Flow cytometry was performed as described.

### T cell mediated killing of Mtb by macrophages

T cells from the spleens of Mtb-infected mice were purified via negative selection, using biotinylated antibodies toward B220 and CD11b and Dynabeads (Invitrogen). Bone marrow-derived macrophages (BMDM) were derived from mouse bone marrow progenitors grown in 20% L929-conditioned cell culture media for 7 days, then *in vitro* infected with Mtb at MOI 1. 2x10^5^ Purified effector T cells were incubated with 1x10^5^ infected BMDC in antibiotic free media supplemented with 20% L929-conditioned media for 6 days. On day 6, cells were lysed and the supernatant plated on Middlebrook 7H11 agar plates (BD, Franklin Lakes, NJ) for CFU determination.

### Statistical analysis

Figures are shown with mean ± SEM. When comparing experimental values from two groups of mice, two-tailed Student's t-tests were used. When comparing experimental values from multiple groups, one-way ANOVA was used. Mortality statistics were calculated using the Log-Rank test. Statistically significant differences are noted (***P < 0.001; **P < 0.01; *P < 0.05). Statistical analysis was performed with GraphPad Prism software (GraphPad, La Jolla, CA).

## Supporting information

S1 Fig Qa-1 presents Mtb peptides to CD8^+^ T effector cells from Mtb-infected B6 mice and induces peptide-specific cytotoxicity. (A) B6 mice were infected with a low-dose of Mtb, with splenic lymphocytes harvested at 4 weeks p.i. and enriched for CD8^+^ T cells. ELISpot assays were performed using enriched CD8^+^ T cells as responders and MHC II^-/-^ BMDC incubated with peptide as antigen presenting cells. Representative of 2 independent experiments. (B) Qa-1^+/+^ and Qa-1^-/-^ BMDC were co-cultured with CD8^+^ T cells enriched as in (A), with either media alone or Mtb peptide. After 24 hours, BMDC were harvested and analyzed by flow cytometry for Annexin V expression. Frequency of Annexin V expression for 3 individual B6 mice is shown normalized to media alone control. Representative of 2 independent experiments.
(TIF)

S2 Fig Bacterial burdens in Qa-1^+/+^ and Qa-1^-/-^ littermate mice during low-dose Mtb infection. Age-matched, sex-matched Qa-1^+/+^ and Qa-1^-/-^ littermates were infected with a low dose of aerosolized Mtb. Spleen and lung were harvested at indicated time points, homogenized, and plated for bacterial burden. Data pooled from at least 2 experiments, n ≥ 7 mice per group per time point.
(TIF)

S3 Fig Cell type recruitment to lung during high-dose Mtb infection. Qa-1^+/+^ and Qa-1^-/-^ littermates were infected with a high-dose of aerosolized Mtb. Lung leukocytes were isolated at 4 weeks p.i. and recruitment of B cells (B220^+^ CD11c^-^), dendritic cells (CD11c^+^), neutrophils (CD11b^+^ Ly6G^+^), NK cells (TCRβ^-^ NK1.1^+^), CD8^+^ T cells (TCRβ^+^ CD8^+^), CD4^+^ T cells (TCRβ^+^ CD4^+^), and Macrophages (Mϕ) (CD11b^+^ F4/80^+^) were analyzed by flow cytometry. Data representative of 2 independent experiments, n ≥ 4 mice per group.
(TIF)

S4 Fig Naive Qa-1^-/-^ and Qa-1^+/+^ mice have similar expression of inhibitory NK markers on T cells. Splenocytes from naïve Qa-1^+/+^ and Qa-1^-/-^ littermates were isolated and analyzed by flow cytometry. The total number of CD8^+^ T cells, CD4^+^ T cells, and NK cells expressing CD94, NKG2A, or Ly49D was determined. n = 2–6, data pooled from 2 independent experiments.
(TIF)

S5 Fig Infected Qa-1^-/-^ and Qa-1^+/+^ mice express comparable levels of NKG2D and very little NKG2C/E. (A) Representative dot plots of surface NKG2A/C/E and NKG2D expression on lung lymphocytes from high-dose infected Qa-1^+/+^ and Qa-1^-/-^ littermates at 4 weeks p.i., as determined by flow cytometry. Data representative of 2 independent experiments, n ≥ 4 mice per group. (B) mRNA was extracted from purified splenic CD8^+^ T cells from high-dose Mtb-infected Qa-1^+/+^ and Qa-1^-/-^ mice at 4 weeks p.i. qPCR was performed on resulting cDNA for NKG2A and NKG2C/E expression levels. NKG2A fold change normalized to NKG2C/E.
(TIF)

S6 Fig NK cells in Qa-1^-/-^ and Qa-1^+/+^ mice have similar functional capacities. Qa-1^+/+^ and Qa-1^-/-^ mice were infected intravenously with 1x10^8^ Mtb bacteria for 24 hours. (A) Splenic lymphocytes were isolated from infected mice and stimulated with PMA/ionomycin for 4 hours. The number of IFN-γ^+^ NK cells in the spleen was determined by intracellular cytokine staining. (B) NK cell cytotoxicity assay was performed by incubating fluorescently labeled YAC-1 target cells and splenic lymphocyte effectors from infected mice at various ratios. Cells were co-cultured for 5 hours, then stained with 7AAD for determination of YAC-1 cell death by flow cytometry. n = 3 mice per group.
(TIF)

S7 Fig Suppressive CD8^+^ T cells unable to be identified by surface phenotype during Mtb infection. Qa-1^+/+^ and Qa-1^-/-^ littermates were infected with a high dose of aerosolized Mtb, and cell surface phenotype of lymphocytes was analyzed by flow cytometry. (A) Number of splenic CD25^+^ FoxP3^+^ CD8^+^ T cells at 4 weeks p.i. (B) Number of splenic CD44^hi^ CD122^+^ Ly49^+^ CD8^+^ T cells at 4 weeks p.i. n = 2–4 mice per group, representative of 2 independent experiments.
(TIF)

S1 Table *Mycobacterium tuberculosis* peptides tested for binding to Qa-1. A panel of HLA-E-binding *Mycobacterium tuberculosis* peptides were generated for testing for binding to Qa-1. Peptides in bold showed relatively high binding to Qa-1 and were used for further experiments. * UniProtKB/Swissprot/EMBL accession number.
(TIF)

S1 File Supplementary materials and methods. (DOCX)
