# Supplementary figures and images for: MHC Ib molecule Qa-1 presents Mycobacterium tuberculosis peptide antigens to CD8+ T cells and contributes to protection against infection
Source: PLoS Pathog. 2017 May 5;13(5):e1006384. doi: 10.1371/journal.ppat.1006384 (PMC5435364; doi:10.1371/journal.ppat.1006384)

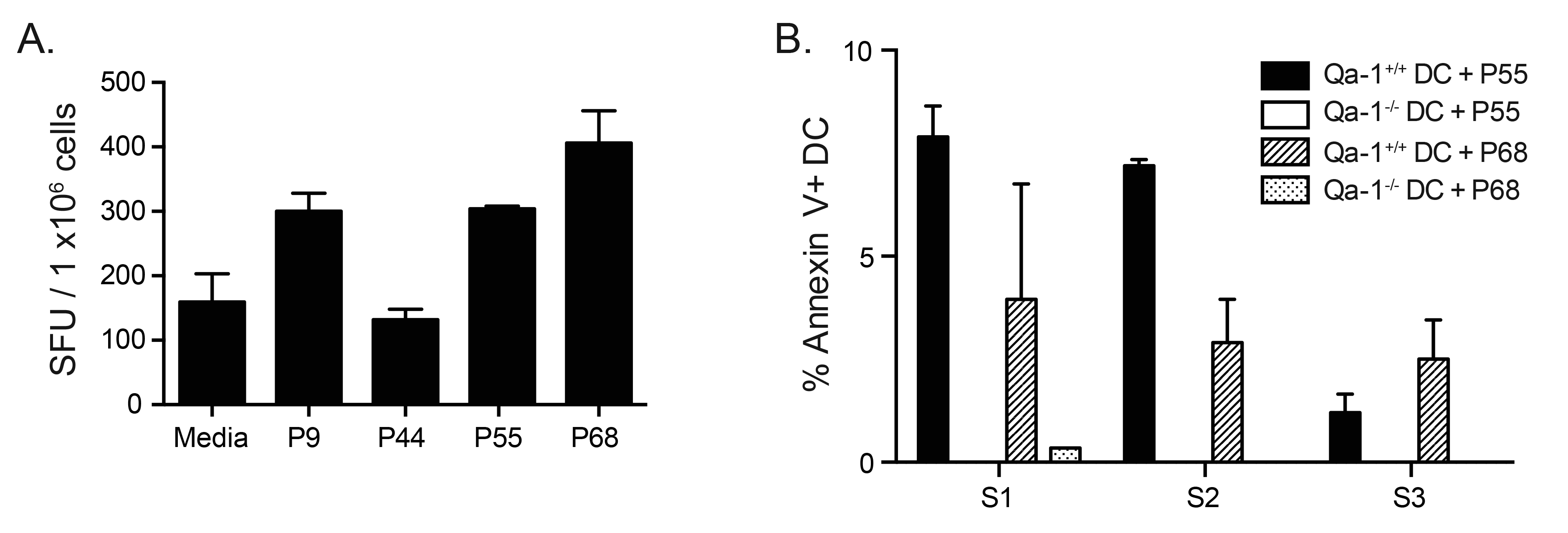

Supplement: S1 Fig — (A) B6 mice were infected with a low-dose of Mtb, with splenic lymphocytes harvested at 4 weeks p.i. and enriched for CD8+ T cells. ELISpot assays were performed using enriched CD8+ T cells as responders and MHC II-/- BMDC incubated with peptide as antigen presenting cells. Representative of 2 independent experiments. (B) Qa-1+/+ and Qa-1-/- BMDC were co-cultured with CD8+ T cells enriched as in (A), with either media alone or Mtb peptide. After 24 hours, BMDC were harvested and analyzed by flow cytometry for Annexin V expression. Frequency of Annexin V expression for 3 individual B6 mice is shown normalized to media alone control. Representative of 2 independent experiments. (TIF) [file ppat.1006384.s001.tif]

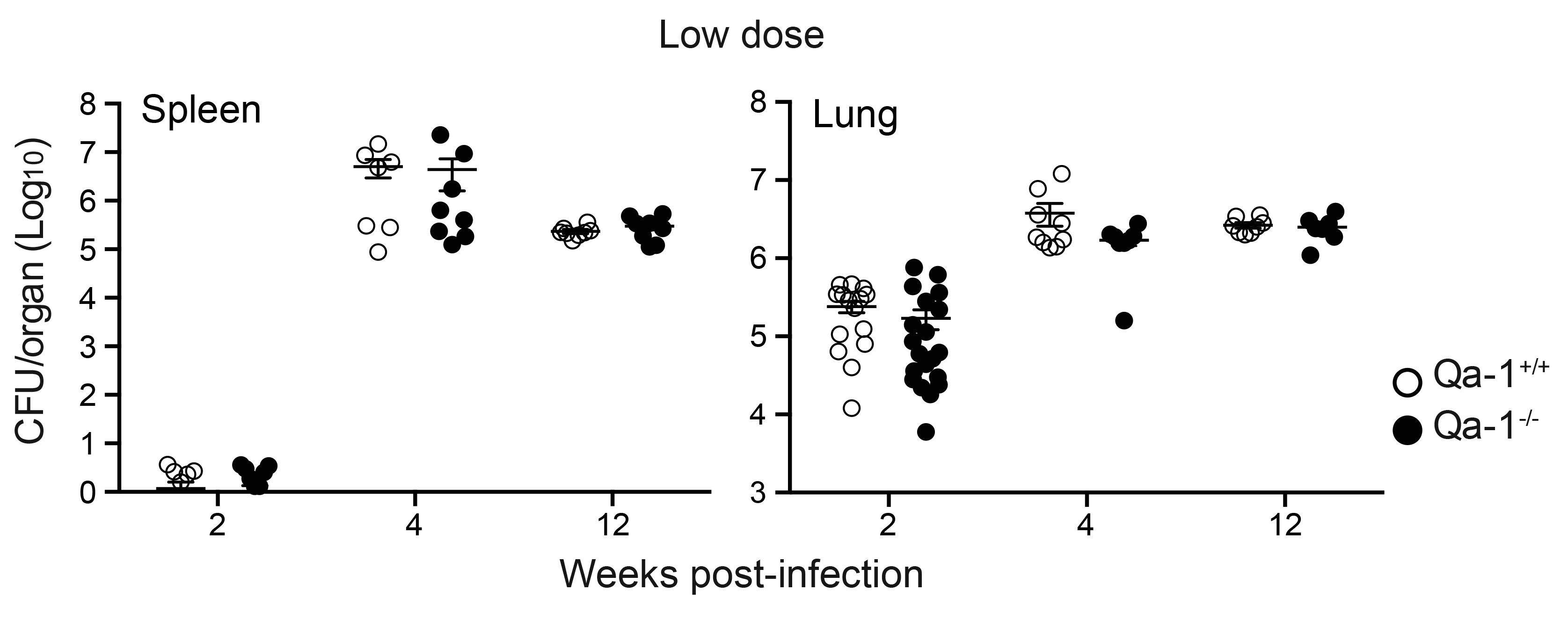

Supplement: S2 Fig — Age-matched, sex-matched Qa-1+/+ and Qa-1-/- littermates were infected with a low dose of aerosolized Mtb. Spleen and lung were harvested at indicated time points, homogenized, and plated for bacterial burden. Data pooled from at least 2 experiments, n ≥ 7 mice per group per time point. (TIF) [file ppat.1006384.s002.tif]

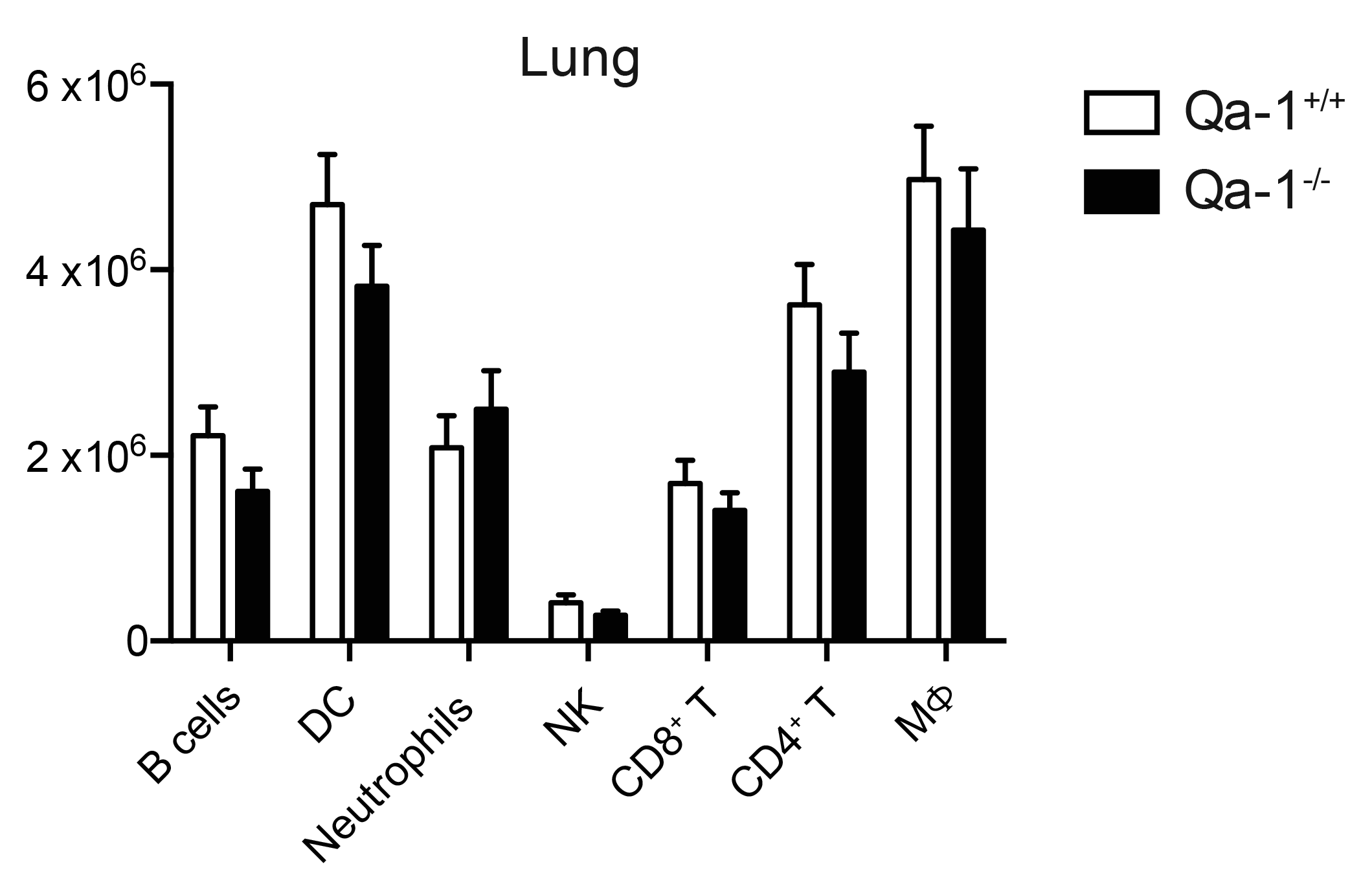

Supplement: S3 Fig — Qa-1+/+ and Qa-1-/- littermates were infected with a high-dose of aerosolized Mtb. Lung leukocytes were isolated at 4 weeks p.i. and recruitment of B cells (B220+ CD11c-), dendritic cells (CD11c+), neutrophils (CD11b+ Ly6G+), NK cells (TCRβ- NK1.1+), CD8+ T cells (TCRβ+ CD8+), CD4+ T cells (TCRβ+ CD4+), and Macrophages (Mϕ) (CD11b+ F4/80+) were analyzed by flow cytometry. Data representative of 2 independent experiments, n ≥ 4 mice per group. (TIF) [file ppat.1006384.s003.tif]

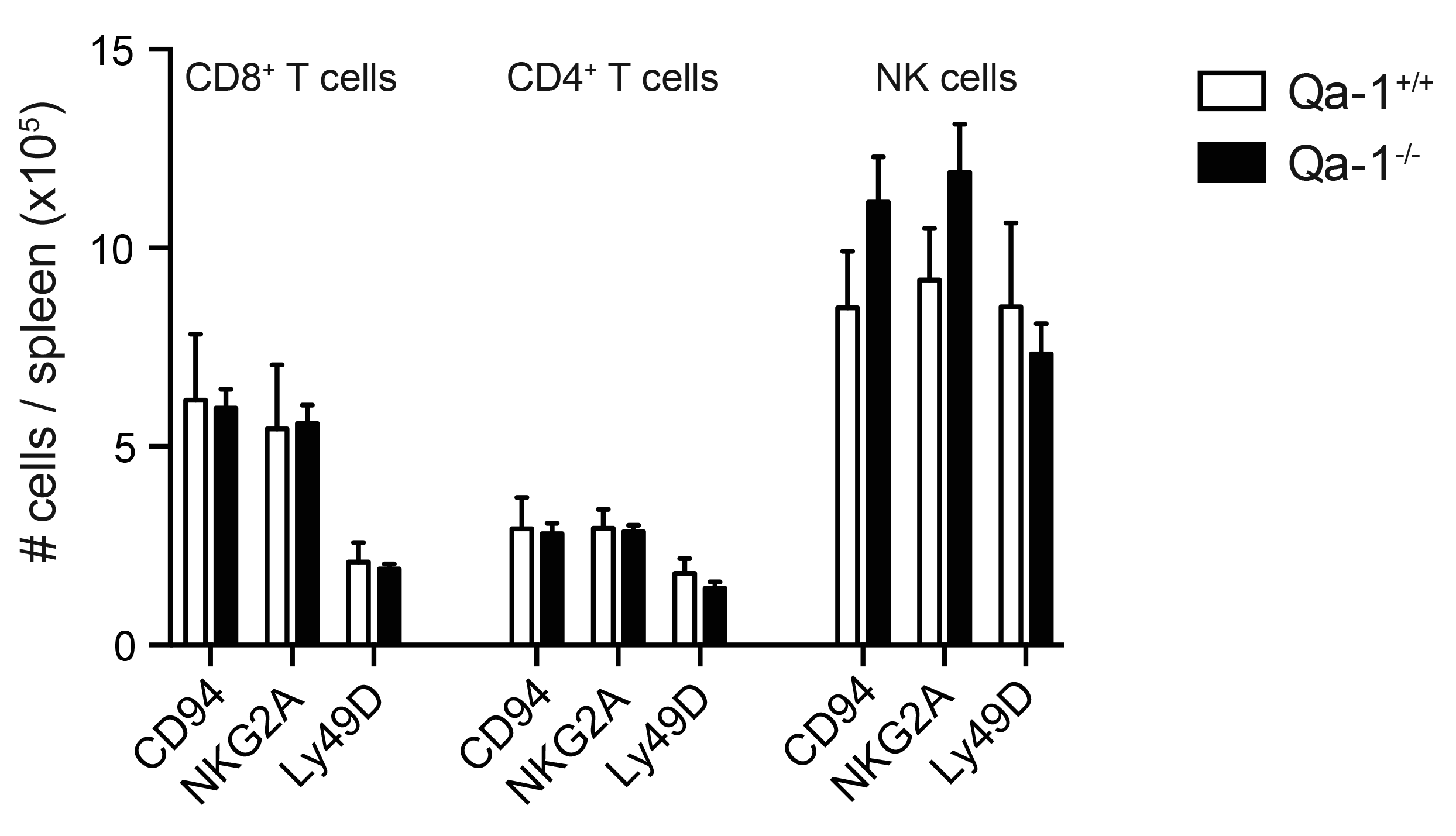

Supplement: S4 Fig — Splenocytes from naïve Qa-1+/+ and Qa-1-/- littermates were isolated and analyzed by flow cytometry. The total number of CD8+ T cells, CD4+ T cells, and NK cells expressing CD94, NKG2A, or Ly49D was determined. n = 2–6, data pooled from 2 independent experiments. (TIF) [file ppat.1006384.s004.tif]

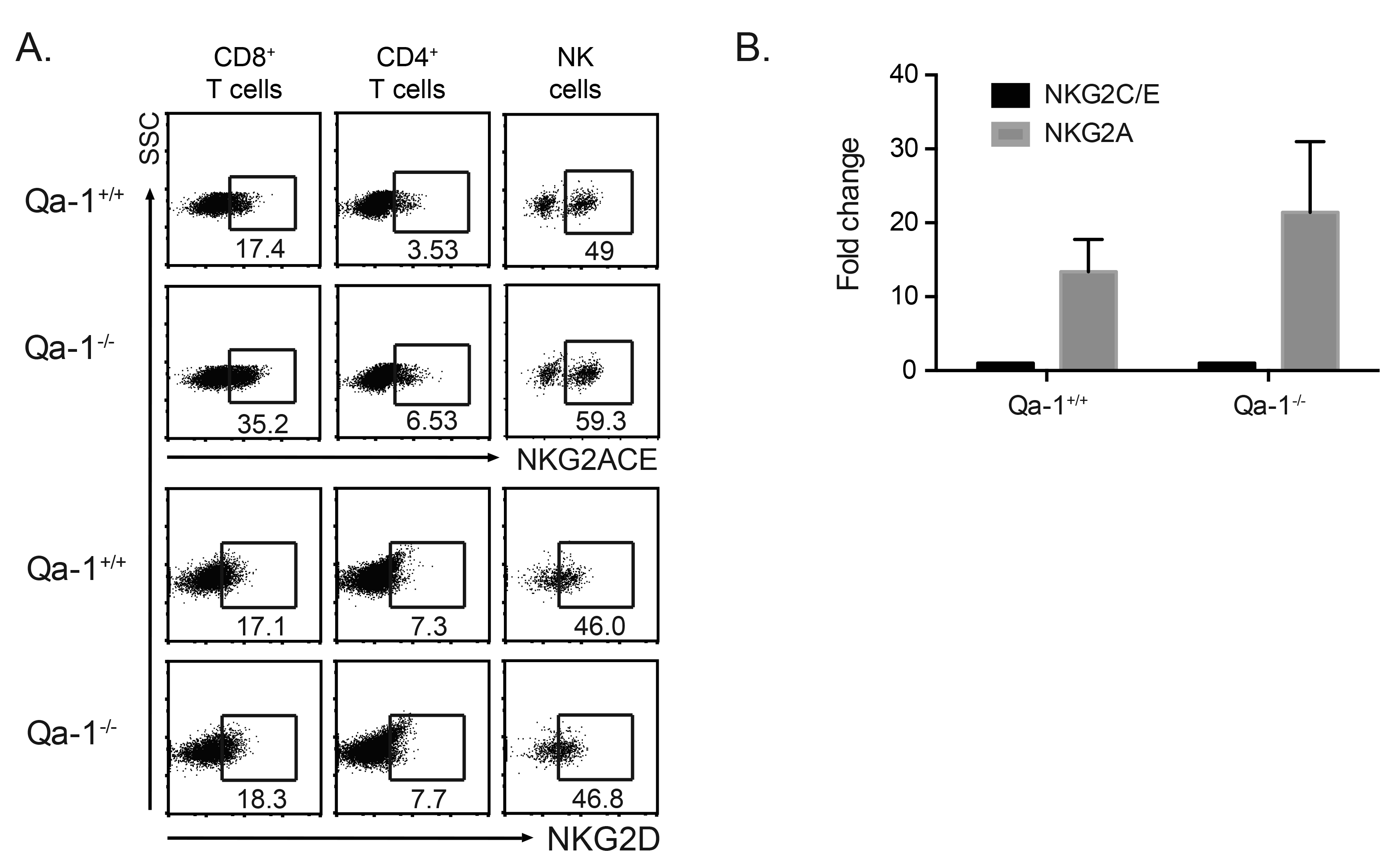

Supplement: S5 Fig — (A) Representative dot plots of surface NKG2A/C/E and NKG2D expression on lung lymphocytes from high-dose infected Qa-1+/+ and Qa-1-/- littermates at 4 weeks p.i., as determined by flow cytometry. Data representative of 2 independent experiments, n ≥ 4 mice per group. (B) mRNA was extracted from purified splenic CD8+ T cells from high-dose Mtb-infected Qa-1+/+ and Qa-1-/- mice at 4 weeks p.i. qPCR was performed on resulting cDNA for NKG2A and NKG2C/E expression levels. NKG2A fold change normalized to NKG2C/E. (TIF) [file ppat.1006384.s005.tif]

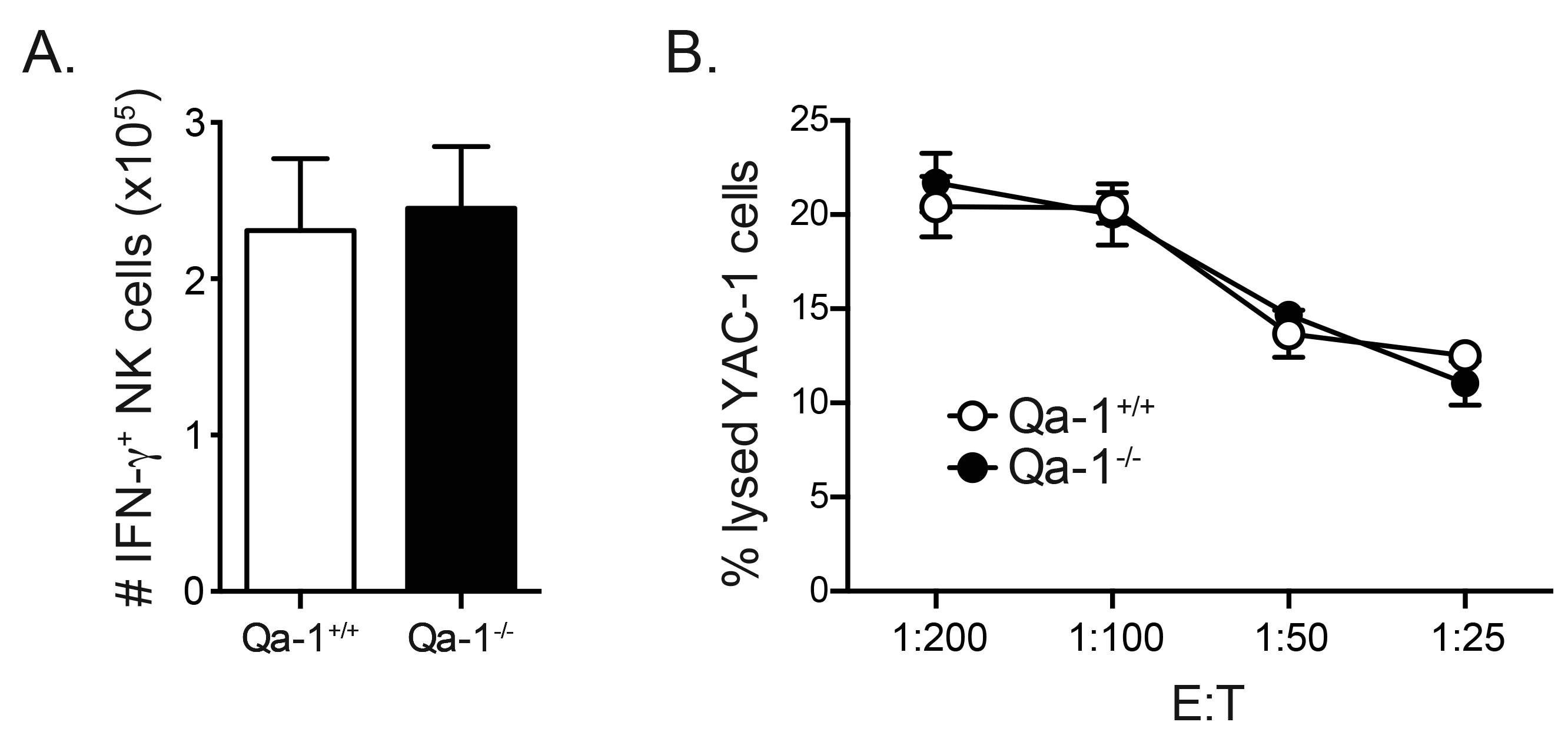

Supplement: S6 Fig — Qa-1+/+ and Qa-1-/- mice were infected intravenously with 1x10^8^ Mtb bacteria for 24 hours. (A) Splenic lymphocytes were isolated from infected mice and stimulated with PMA/ionomycin for 4 hours. The number of IFN-γ+ NK cells in the spleen was determined by intracellular cytokine staining. (B) NK cell cytotoxicity assay was performed by incubating fluorescently labeled YAC-1 target cells and splenic lymphocyte effectors from infected mice at various ratios. Cells were co-cultured for 5 hours, then stained with 7AAD for determination of YAC-1 cell death by flow cytometry. n = 3 mice per group. (TIF) [file ppat.1006384.s006.tif]

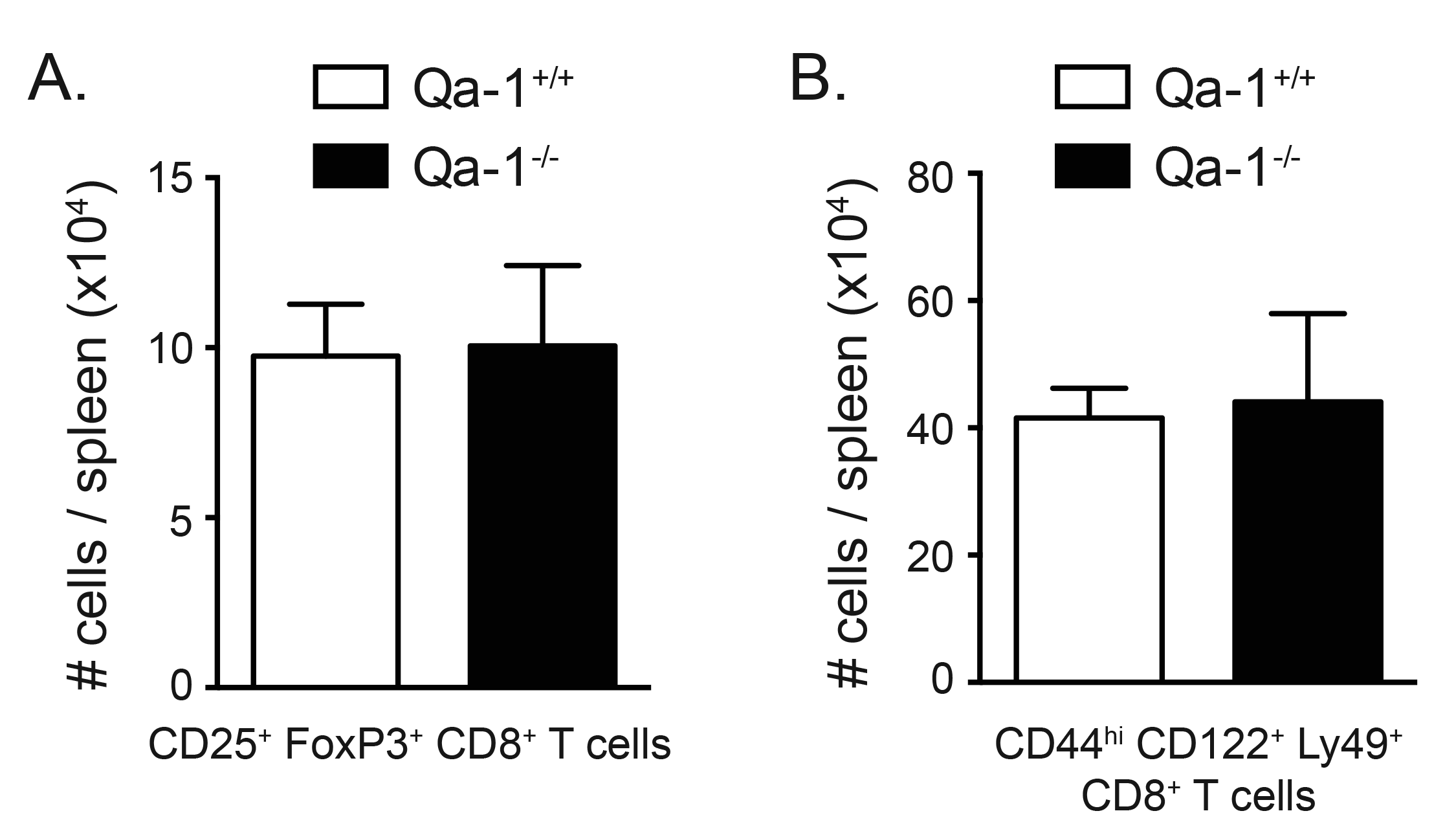

Supplement: S7 Fig — Qa-1+/+ and Qa-1-/- littermates were infected with a high dose of aerosolized Mtb, and cell surface phenotype of lymphocytes was analyzed by flow cytometry. (A) Number of splenic CD25+ FoxP3+ CD8+ T cells at 4 weeks p.i. (B) Number of splenic CD44hi CD122+ Ly49+ CD8+ T cells at 4 weeks p.i. n = 2–4 mice per group, representative of 2 independent experiments. (TIF) [file ppat.1006384.s007.tif]

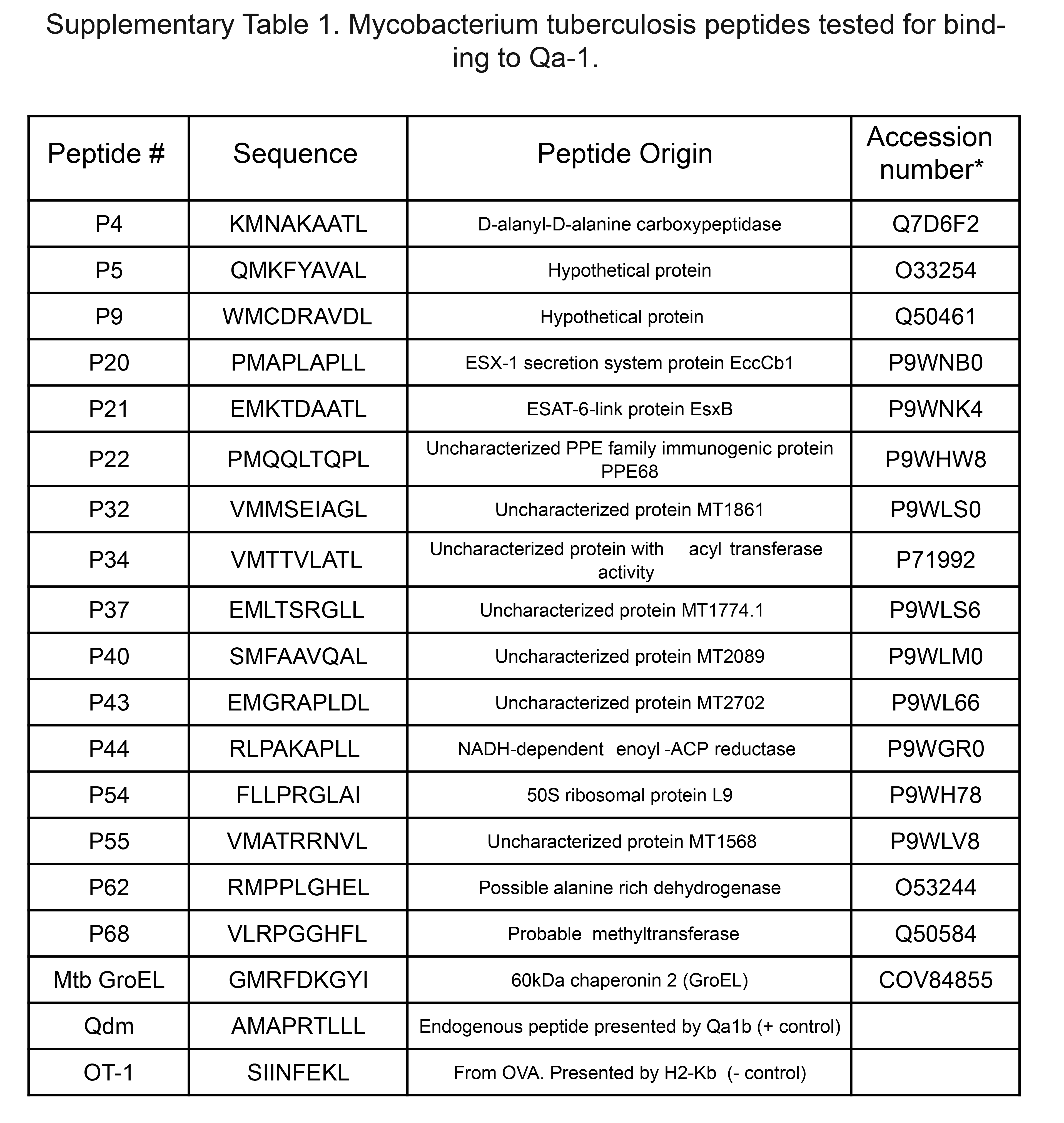

Supplement: S1 Table — A panel of HLA-E-binding Mycobacterium tuberculosis peptides were generated for testing for binding to Qa-1. Peptides in bold showed relatively high binding to Qa-1 and were used for further experiments. * UniProtKB/Swissprot/EMBL accession number. (TIF) [file ppat.1006384.s008.tif]
