# Supplementary material for: MHC Ib molecule Qa-1 presents Mycobacterium tuberculosis peptide antigens to CD8+ T cells and contributes to protection against infection
Source: PLoS Pathog. 2017 May 5;13(5):e1006384. doi: 10.1371/journal.ppat.1006384 (PMC5435364; doi:10.1371/journal.ppat.1006384)
Supplement: S1 File — (DOCX) [file ppat.1006384.s009.docx]

**Supplementary Materials and Methods**

**Mtb peptide-specific cytotoxicity assay**

Qa-1^+/+^ or Qa-1^-/-^ BMDCs were fluorescently labeled (CellTrace Cell Proliferation Kit, Invitrogen, Carlsbad, CA) and incubated with M5/114 supernatant (anti-MHC II) and either 5 μM of Mtb peptide or media. Splenic CD8^+^ T cells from low-dose Mtb-infected mice were enriched as described above and co-cultured with BMDCs at an effector to target ratio of 4:1, in duplicate. After 24h incubation at 37°C, BMDC were harvested using trypsin digestion and analyzed by flow cytometry for Annexin V (BioLegend, San Diego, CA) expression.

**NK cell cytokine production and cytotoxicity assay**

Short-term, high dose i.v. Mtb infection for analysis of NK cell function was performed as described [47]. Briefly, frozen aliquots of Mtb H37Rv were thawed and diluted in PBS with 0.05% Tween 80. Mice were infected with Mtb at 1x10^8^ CFU/mouse i.v. via tail vein for 24 hours. For IFN-γ production assay, splenocytes from infected animals were harvested and incubated with PMA (20 ng/ml) and ionomycin (500 ng/ml) for 30 min. Monensin (5 μM) was then added and lymphocytes were incubated for an additional 4 hours. Cells were then stained for cell surface markers, fixed with 4% paraformaldehyde, permeabilized with 0.2% saponin, and then stained with FITC-conjugated anti-IFN-γ (BioLegend, San Diego, CA). For NK cell cytotoxicity assay, splenic lymphocytes from infected animals were incubated with fluorescently labeled YAC-1 target cells at different E:T ratios for 5 hours. Cells were then stained for 7AAD (eBioscience, San Diego, CA) per manufacturer’s instructions. Flow cytometry was performed as described.

**Quantitative RT-PCR**

Total RNA from purified CD8^+^ and CD4^+^ T cells was extracted using Trizol reagent (Invitrogen, Carlsbad, CA), with first-strand cDNA synthesis performed using Superscript III reverse transcriptase (Invitrogen, Carlsbad, CA) according to the manufacturer’s instructions. Real-time PCR was performed using an iQ5 instrument (Bio-Rad, Hercules, CA). Gene transcripts were normalized to HPRT mRNA. Primers used: NKG2A For (ACTCATTGCTGGTACCCTGGG), NKG2A Rev (GAGGACAAGGCTGTGCTGAAG), NKG2C/E For (ACCTGCTTGGAACTGAACAGG), NKG2C/E Rev (GCAAAATTTTTGCAGTAGCCATG).
